# High-throughput analysis of chemical components and theoretical ethanol yield of dedicated bioenergy sorghum using dual-optimized partial least squares calibration models

**DOI:** 10.1186/s13068-017-0892-z

**Published:** 2017-09-04

**Authors:** Meng Li, Jun Wang, Fu Du, Boubacar Diallo, Guang Hui Xie

**Affiliations:** 1 College of Agronomy and Biotechnology, China Agricultural University, Beijing, 100193 China; 2 National Energy R&D Center for Non-food Biomass, China Agricultural University, Beijing, 100193 China

**Keywords:** Bioenergy sorghum, Near infrared spectroscopy, Linear discriminant analysis model, Chemical components, Theoretical ethanol yield, Optimal sample subset partitioning, Optimal variable selection, Partial least squares model

## Abstract

**Background:**

Due to its chemical composition and abundance, lignocellulosic biomass is an attractive feedstock source for global bioenergy production. However, chemical composition variations interfere with the success of any single methodology for efficient bioenergy extraction from diverse lignocellulosic biomass sources. Although chemical component distributions could guide process design, they are difficult to obtain and vary widely among lignocellulosic biomass types. Therefore, expensive and laborious “one-size-fits-all” processes are still widely used. Here, a non-destructive and rapid analytical technology, near-infrared spectroscopy (NIRS) coupled with multivariate calibration, shows promise for addressing these challenges. Recent advances in molecular spectroscopy analysis have led to methodologies for dual-optimized NIRS using sample subset partitioning and variable selection, which could significantly enhance the robustness and accuracy of partial least squares (PLS) calibration models. Using this methodology, chemical components and theoretical ethanol yield (TEY) values were determined for 70 sweet and 77 biomass sorghum samples from six sweet and six biomass sorghum varieties grown in 2013 and 2014 at two study sites in northern China.

**Results:**

Chemical components and TEY of the 147 bioenergy sorghum samples were initially analyzed and compared using wet chemistry methods. Based on linear discriminant analysis, a correct classification assignment rate (either sweet or biomass type) of 99.3% was obtained using 20 principal components. Next, detailed statistical analysis demonstrated that partial optimization using sample set partitioning based on joint *X*–*Y* distances (SPXY) for sample subset partitioning enhanced the robustness and accuracy of PLS calibration models. Finally, comparisons between five dual-optimized strategies indicated that competitive adaptive reweighted sampling coupled with the SPXY (CARS-SPXY) was the most efficient and effective method for improving predictive performance of PLS multivariate calibrations.

**Conclusions:**

As a dual-optimized methodology, sample subset partitioning combined with variable selection is an efficient and straightforward strategy to enhance the accuracy and robustness of NIRS models. This knowledge should facilitate generation of improved lignocellulosic biomass feedstocks for bioethanol production. Moreover, methods described here should have wider applicability for use with feedstocks incorporating multispecies biomass resource streams.

**Electronic supplementary material:**

The online version of this article (doi:10.1186/s13068-017-0892-z) contains supplementary material, which is available to authorized users.

## Background

Interest in biomass as an alternative energy resource has been increasing dramatically in step with increases in fossil energy costs worldwide [1]. As a promising advanced biofuel, bioethanol has become one of the most practical solutions for carbon emission reduction and food security in recent decades [2, 3]. Moreover, production of second-generation bioethanol derived from lignocellulosic agricultural wastes offers farmers and rural communities more economic opportunities than did first-generation bioethanol production [4]. Consequently, optimization of lignocellulosic feedstocks for second-generation bioethanol production is currently under intensive development globally [5]. Among candidate biofuel feedstocks, sorghum (*Sorghum bicolor* L.) contains exceptional levels of soluble sugars (in the stem) and starch (in the grain), both of which are directly fermentable into bioethanol [1, 6]. Meanwhile, a large amount of degradable lignocellulose from stem is available for second-generation bioethanol production after the harvest season. As a typical C4 crop, sorghum offers several advantages: rapid growth rate, high tolerance to drought and saline/alkaline soil conditions, and worldwide adaptability. Nowadays, sorghum is the fifth most widely produced cereal crop in the world and is grown not only for grain production but also for fiber, forage, and sugar production [7, 8]. For energy purposes, sorghum may be divided into two specific categories: sweet sorghum and biomass sorghum. Sweet sorghum accumulates high levels of sugar in the stem of the plant, while biomass sorghum contains abundant structural carbohydrates that are produced in sufficient quantities to meet future energy demands [8]. To date, the application of sorghum to bioethanol production has rarely been reported. Therefore, the first essential step would be to compare energy potentials between these two sorghum categories.

Bioenergy sorghum cell walls consist mainly of cellulose, hemicellulose, lignin, and ash. Cellulose, which consists of β-1,4 linked linear glucans, is the most abundant biopolymer and carbon sink on earth [9]. Hemicellulose is a class of heterogeneous polysaccharides that constitutes between 15 and 35% of the total biomass of hardwoods and herbaceous plants [10]. Lignin is a hydrophobic polymer consisting of three major phenolic components: hydroxyphenyl, guaiacyl, and syringyl units [11]. Ash-forming elements are often embedded in biomass and include potassium, calcium, magnesium, silicon, sodium, phosphorus, sulfur, and chlorine. Knowledge of both ash level and composition are prerequisites for effective industrial biomass process design [12, 13], but these parameters are difficult to determine. As a consequence, the diversity of chemical components and their varied proportions among lignocellulosic feedstocks frequently leads to the so-called “biomass recalcitrance,” requiring expensive and time-consuming bioenergy extraction processing steps [14]. Therefore, high-throughput determinations of chemical composition and theoretical ethanol yield (TEY) are increasingly necessary in order to screen large numbers of lignocellulosic feedstocks. Successful screening will guide development of plant breeding and genetic modification programs toward the ultimate goal of achieving high biofuel yields at low cost [4].

Near-infrared spectroscopy (NIRS) technology is based on the absorption of electromagnetic radiation by chemical covalent bonds (primarily C–H, N–H, O–H, and S–H) combined with multivariate calibration [15]. As a non-destructive and rapid analytical method, NIRS has been widely used for qualitative analysis of various organic feedstocks and for physical and chemical quantitative analyses across the food, pharmaceutical, and agricultural industries [16, 17]. With regard to qualitative analysis, numerous promising techniques for necessary preprocessing of NIRS data have been developed during the last few decades including principal component analysis, K-nearest neighbor analysis, genetic algorithms, partial least squares discriminant analysis, and others [5, 18]. As part of these analyses, linear discriminant analysis is a well-known and frequently used computational tool [15, 19]. In general, bioenergy extraction processes are sensitive to defined distributions of physical and chemical characteristics, e.g., “Biomass Variability” or “Feedstock Quality,” that vary greatly among plant species impact biofuel conversion performance [20, 21]. As an ideal high-throughput biomass classification technique, NIRS qualitative analysis has been viewed as increasingly valuable to the bioenergy industry [5, 13, 22, 23]. However, very little research has yet been done to evaluate NIRS for lignocellulosic biomass feedstock classification in the preparation for bioenergy production from sorghum [1, 4, 6, 23–26].

For quantitative analysis in the bioenergy sector, earlier research mainly focused on the development of spectral preprocessing steps and multivariate calibration algorithms [22, 23]. As two crucial quantitative modeling steps, sample subset partitioning and spectra variable selection have offered great advantages for improving the accuracy and robustness of prediction models [27–30]. On one hand, the predictive performance of the NIRS model heavily depends on the internal connection between spectral features and various analytical properties of the sample subset [28, 30, 31]. In this context, several studies have addressed the problem of sample subset partitioning. Galvão et al. [32] reported a stepwise procedure for selecting samples according to their differences in both *x* (instrumental responses) and *y* (predicted parameter) spaces. Subsequently, the use of a method incorporating sample set partitioning based on joint *X*–*Y* distances (SPXY) has gained wide acceptance as an advantageous alternative to existing sample subset partitioning strategies [28, 32]. On the other hand, the selection of spectral variables allows for the selection of the optimal variables subset that would greatly improve prediction performance by improving calibration reliability prior to inverse calibration (for primarily PLS). Because PLS does not completely solve the over-fitting problem observed in multivariate calibration and pattern recognition without variable selection [29], the following efficient variable selection methods have been devised: competitive adaptive reweighted sampling, selectivity ratio, variable importance for projection, Monte Carlo-uninformative variable elimination, and uninformative variable elimination [33–37]. Neither sample subset partitioning nor spectra variable selection had exhibited enough promise in previous studies to encourage their use for the determination of lignocellulosic properties [5, 13, 22, 23, 38]. Therefore, from a dual-optimization standpoint, it appears essential to identify the enhancement of both sample subset partitioning and variable selection that affects the performance of NIRS predictive models.

In the present study, we made comparisons between 70 sweet sorghum and 77 biomass sorghum samples by focusing on the main chemical components and bioethanol potentials of stem. Next, a reliable and accurate qualitative method was presented for lignocellulosic feedstock classification into sweet or biomass category using linear discriminant analysis. SPXY was then employed to partition calibration and validation subsets for 147 bioenergy sorghum samples. Next, six partial-optimized PLS models were developed for the prediction of chemical components and TEY. Ultimately, the main objective of this study was to compare five dual-optimized strategies for improving the predictive performance of PLS multivariate calibrations. In summary, this work provides powerful qualitative and quantitative tools to guide future feedstock selection, bioenergy crop breeding, and genetic modification programs to achieve more efficient biofuel production from diverse lignocellulosic feedstocks.

## Results and discussion

### Diversity of soluble sugar, cell wall components, and TEY

**Fig. 1:**
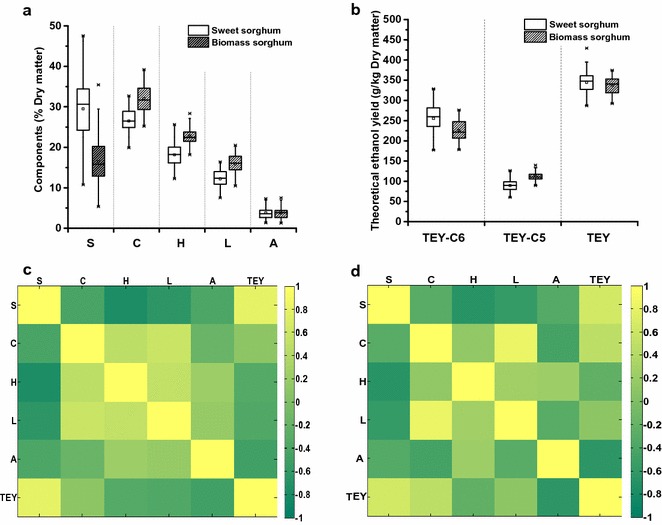
Diversity of chemical components and theoretical ethanol yield, and their correlations in 147 bioenergy sorghums. *S* soluble sugar, *C* cellulose, *H* hemicellulose, *L* lignin, *A* ash, *TEY* theoretical ethanol yield, *TEY-C6* TEY from hexose, and *TEY-C5* TEY from pentose. **a** Chemical components of 147 bioenergy sorghum samples, **b** TEY of 147 bioenergy sorghum samples, **c** correlations among chemical components and TEY of 70 sweet sorghum samples, **d** correlations among chemical components and TEY of 77 biomass sorghum samples

Sample diversity was reflected by the levels of soluble sugar, cellulose, hemicellulose, lignin, ash, and TEY shown in Fig. 1. In general, sweet sorghum samples exhibited much higher soluble sugar levels (10.8–47.6%) than did biomass sorghum samples (5.4–35.4%) (Fig. 1a; Additional file 1: Table A1). Conversely, biomass sorghum samples were relatively richer in cellulose, hemicellulose, and lignin. However, both sorghum categories displayed almost the same ash level, averaging 3.8%, which was relatively lower than that observed in other bioenergy crops [13]. To confirm the energy biofuel potential of bioenergy sorghum stem, we further carried out TEY originating from both hexose (C6) and pentose (C5) in this work (Fig. 1b; Additional file 1: Table A2). Due to the high levels of soluble sugar, sweet sorghum exhibited relatively higher TEY from C6 than did biomass sorghum, with the average value of 255.3 g/kg. By contrast, biomass sorghum exhibited more TEY from C5, with the average value of 112.1 g/kg. As a consequence, the total TEY levels of both sorghum categories were nearly the same. In particular, the total TEY of all 147 bioenergy sorghum samples ranged from 287.1 to 429.3 g/kg, with an average value of 340.9 g/kg. These results were similar to that of sorghum grain (312.0 g/kg) and show that bioenergy sorghum stem is a viable biofuel feedstock [1].

Because representative bioenergy sorghum samples displayed large population variability and broad distributions of sample characteristics, we further performed correlation analyses between chemical components and TEY values (Fig. 1c, d). Notably, a significantly positive correlation was observed between soluble sugar and TEY in both sweet sorghum and biomass sorghum samples (*p* < 0.01), indicating that soluble sugar greatly contributes to TEY in bioenergy sorghum. A high level of cellulose also exhibited a significantly positive correlation (*p* < 0.01) with TEY. Conversely, the hemicellulose level negatively correlated with TEY in the present work. Meanwhile, it was found that lignin and ash, known to contribute to biomass recalcitrance, strongly and negatively were correlated with TEY. Generally speaking, a higher hemicellulose level could generate more bioethanol. However, it exhibited significantly positive correlations with bioconversion barriers (e.g., ash and lignin) and negative correlations with soluble and insoluble carbohydrates in bioenergy sorghum. Therefore, reduction in lignin and/or ash contents could greatly enhance bioethanol potential. Moreover, it indicated to select ideal bioenergy sorghum varieties with relatively lower hemicellulose level, which could lead to higher cellulose and soluble sugar levels for producing a higher TEY level.

### Diversity of NIRS and qualitative classification of bioenergy sorghum samples

**Fig. 2:**
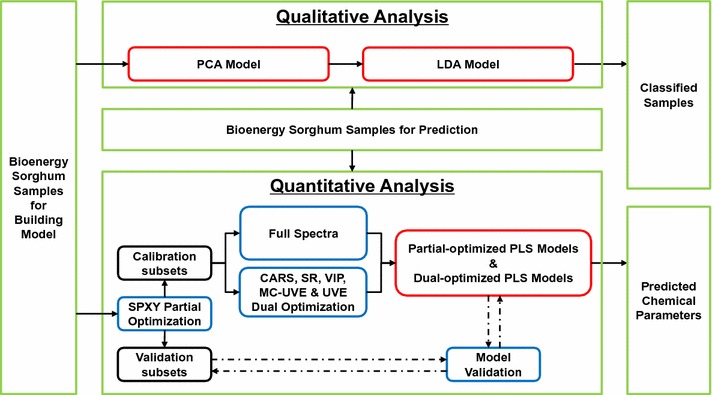
Flowchart of NIRS qualitative and quantitative analyses. *PCA* principal component analysis, *LDA* linear discriminant analysis, *SPXY* sample set partitioning based on joint *X*–*Y* distances, *CARS* competitive adaptive reweighted sampling, *SR* selectivity ratio, *VIP* variable importance for projection, *UVE* uninformative variable elimination, *MC-UVE* UVE couple with the principle of Monte Carlo, *PLS* partial least squares

**Fig. 3:**
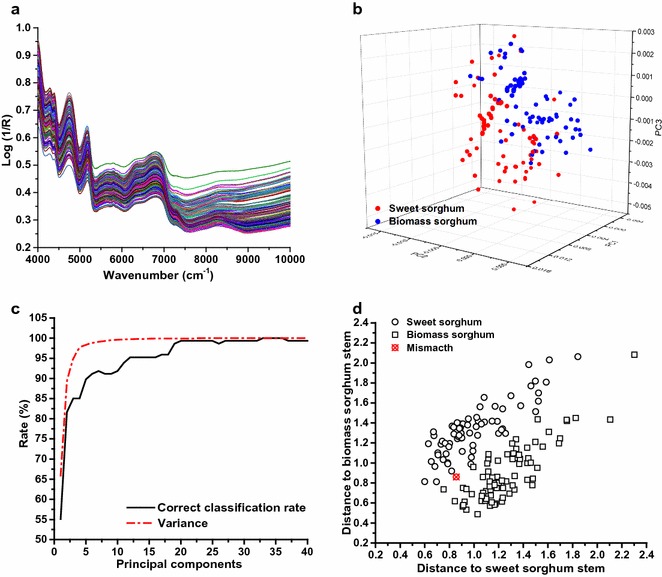
Description and qualitative classification of 147 bioenergy sorghum samples. **a** Original spectra of 147 bioenergy sorghum samples, **b** 3D plot of the principal component analysis scores of 147 bioenergy sorghum samples, **c** the correct classification rate and variance obtained by linear discriminant analysis, **d** Mahalanobis distance of 147 bioenergy sorghum samples (principal components = 20)

A briefly step-by-step flowchart for understanding the high-throughput qualitative and quantitative methodology is available in Fig. 2. Firstly, NIRS of 147 bioenergy sorghum samples was performed using wave numbers from 4000 to 10,000 with a resolution of 8 cm^−1^ (Fig. 3a). The main absorption band peaks occurred in the range from 4000 to 7400 cm^−1^ (Table 1). In previous studies, the strong peak at approximately 5150–5195 cm^−1^ was primarily attributed to O–H asymmetric stretching and O–H deformation bands of water [4, 6, 24]. For soluble sugar, the most important spectral regions were 5150–5195, 5776–5796, and 6775–6822 cm^−1^ [4]. The band deformation (O–H, C–H, and C–H_2_), band stretching vibration (O–H, C–H, C–H_2_, C–O, and C–C), and first overtone stretching (O–H) of cellulose greatly contributed to the absorption band peaks around 4015–4022, 4392–4412, 4760–4780, 5776–5796, 6329–6336, 6775–6822, and 7305–7328 cm^−1^ [4, 38]. The C–H and C–H_2_ deformation and stretching vibration, and the first overtone O–H stretching band of hemicellulose were indicated by spectral changes within wavenumber ranges of 4285–4296, 4392–4412, and 6775–6822 cm^−1^ [4, 38]. Lignin could be identified at 4015–4022, 4392–4412, and 5776–5796 cm^−1^ by its stretching vibration (O–H, C–H, C–O, and C–C) and the overtone stretching band of O–H [24, 38]. Ash, an inorganic component, could not be detected directly using NIRS, but could be determined from its association with the organic component of bioenergy sorghum [13, 39]. Finally, as previously reported, TEY could be calibrated and predicted using absorption band peaks at around 7060, 5230, 4440, and 4330 cm^−1^ [4].

**Table 1:** The main absorption band peak location of chemical components in bioenergy sorghum

Wavenumber (cm^−1^)	Component	Bond vibration
4015–4022	C, L	C–H_str_, C–C_str_
4285–4296	H	C–H_str_, C–H_def_
4392–4412	C, H, L	C–H_str_, C–H_2str_, O–H_str_, C–O_str_, C–H_def_, C–H_2def_
4760–4780	C	O–H_def_, C–H_def_, O–H_str_
5150–5195	W, S	O–H_asym_, O–H_str_, O–H_def_
5776–5796	S, C, L	1st OT C–H_str_
6329–6336	C	1st OT C–H_str_
6775–6822	S, C, H	1st OT C–H_str_
7305–7328	C	1st OT C–H_str_, C–H_def_

*C* cellulose, *H* hemicellulose, *L* lignin, *S* soluble sugar, *W* water

Based on representative reconstructed spectra variables, principal component analysis was primarily developed to identify outliers, as well as for sample comparison and classification [5]. As shown in Fig. 3b, we initially constructed a principal component analysis model whereby the 3D score plot of NIRS from 147 bioenergy sorghum samples displayed a relatively diverse and symmetrical distribution. The score points of sweet sorghum and biomass sorghum samples were not distinctly separated using three principal components (accounting for 95.9% of spectra variance), consistent with a previous bamboo compositional prediction study [24]. Using further qualitative analysis, linear discriminant analysis, which is based on principal component analysis and Mahalanobis distances, was employed to differentiate between the two categories of bioenergy sorghum samples in this study. Figure 3c shows the correct classification rate and variance of the total sample set after considering a maximum number of 40 principal components. Generally, both correct classification rate and variance increased sharply in step with increases observed for principal components (from 0 to 20), before reaching peaks at 99.3 and 100%, respectively. Meanwhile, Fig. 3d demonstrates the Mahalanobis distance from each sample to the models for sweet sorghum and biomass sorghum. It was obvious that only one sample was mismatched while 20 principal components were taken into account. Therefore, the results demonstrate that linear discriminant analysis could be a powerful and efficient tool for biomass feedstock classification for optimization of bioenergy processing [15]. In addition, the broad range of high absorbance and symmetrical distribution of score points indicated that the spectra of bioenergy sorghum samples possessed good sample representation and functioned well during calibration and prediction.

### Optimization of calibration and validation subset partitioning by SPXY

It is well known that the accuracy and robustness of NIRS quantitative analysis models rely heavily on calibration and validation subsets [30]. In this case, a representative calibration subset must be selected on the basis of NIRS and analytical properties that were extracted from a pool of real samples [32]. Meanwhile, representative external validation samples should also be selected to assess the quality of the quantitative analysis model [13]. Nonetheless, studies of sample subset partitioning have been largely overlooked in the bioenergy sector [5, 22, 23, 38]. In recent years, many strategies have been developed to address this problem from an optimization perspective, such as random sampling and Kennard–Stone. Random sampling is a popular technique because of its simplicity. But it does not guarantee the representativity of the sample subset, nor does it prevent extrapolation problems. To solve these problems, Kennard–Stone was developed to cover the multidimensional space in a uniform manner by maximizing the Euclidean distances between the instrumental response vectors (*x*) of the selected samples. However, the predictive performance of the NIRS model heavily depends on the internal connection between instrumental response vectors (*x*) and various analytical properties (*y*) of the sample subset. Hence, a uniquely advantageous method, designated SPXY, was initially employed to sort 147 bioenergy sorghum samples into calibration and validation subsets for multivariate calibration in the present paper. Notably, this method extends the Kennard–Stone algorithm by encompassing the variability in both the *x* and *y* dimensions for the calculation of inter-sample distances [31]. As a consequence, based on the value of predicted objects, one of every four samples was included in the validation subset, while the remaining samples were used for the calibration subset.

**Fig. 4:**
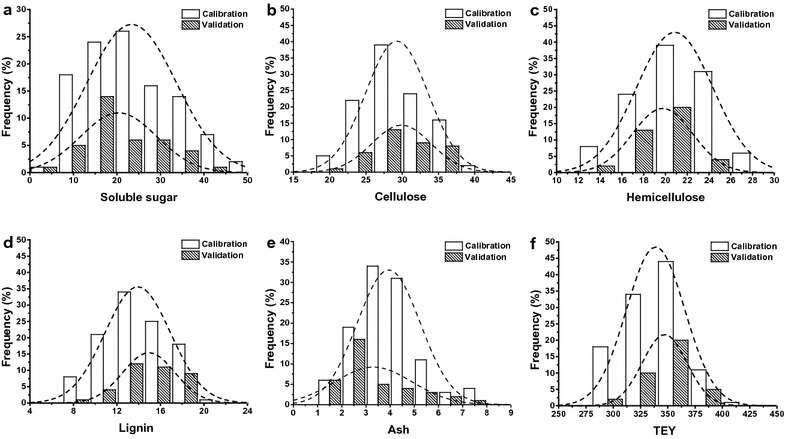
Histograms of chemical components and theoretical ethanol yield. Soluble sugar (**a**), cellulose (**b**), hemicellulose (**c**), lignin (**d**), ash (**e**), and theoretical ethanol yield (**f**) for calibration and external validation subsets that were partitioned by sample set partitioning based on joint *X*–*Y* distances

**Fig. 5:**
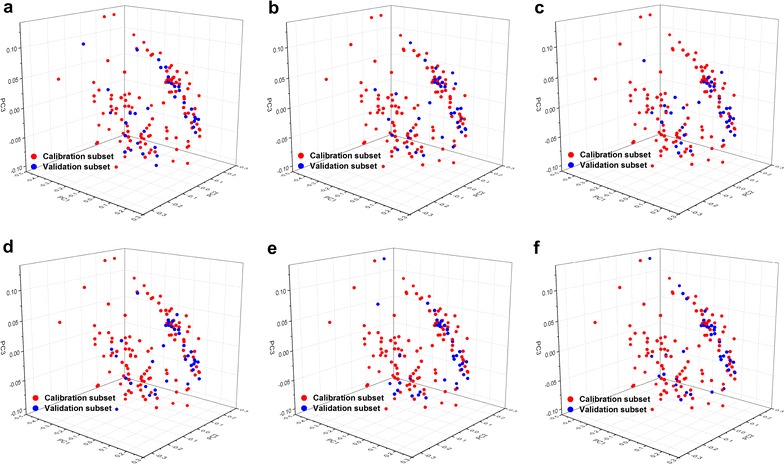
Principal component analysis plots distribution of chemical components and theoretical ethanol yield. Soluble sugar (**a**), cellulose (**b**), hemicellulose (**c**), lignin (**d**), ash (**e**), and theoretical ethanol yield (**f**) for calibration and external validation subsets that partitioned by sample set partitioning based on joint *X*–*Y *distances

As shown in Fig. 4, histograms of calibration and validation subsets for soluble sugar, cellulose, hemicellulose, lignin, ash, and TEY are presented. The dashed lines overlaid upon each histogram represent normal distributions and were used to embody the discrepancy between each histogram and normality. In general, all subsets optimized using SPXY displayed a relatively wide and symmetrical distribution. Despite the fact that the 147 bioenergy sorghum samples originated from a multispecies feedstock population that included various genotypes and phenotypes, almost all the calibration and validation subsets for each property exhibited relatively wide and symmetrical distributions in the present study (Figs. 3a, b, 4). Additionally, each calibration subset represented nearly the same distributions as that of the corresponding validation subset; no distinct bimodal, skewed, or uniform distributions were observed (Fig. 4). In order to further demonstrate the effect of optimization of SPXY on calibration and validation subset partitioning, six principal component analysis models were established for soluble sugar, cellulose, hemicellulose, lignin, ash, and TEY (Fig. 5). Similarly, the 3D score plots of NIRS from both calibration and validation subsets were well mixed and displayed relatively symmetrical distributions. Hence, these results indicate that the SPXY method could efficiently optimize NIRS results as well as the analytical properties of both the calibration subset and related validation subset. These optimizations thus led to an accurate and robust NIRS quantitative analysis model.

### Partial-optimized PLS models

Based on the SPXY-optimized calibration and validation subsets, six partial-optimized PLS models were developed using full spectra for the prediction of soluble sugar, cellulose, hemicellulose, lignin, ash, and TEY. To reduce any multiplicative and additive effects resulting from instrument settings or variations stemming from sample and environmental conditions, the spectra averaged from 64 scans were subjected stepwise to multiplicative scatter correction, then Norris derivative filtering, then first derivative analysis. In addition, all six models were fully cross-validated using the “leave-one-out” method and the optimal number of principal components (from 6 to 9) for each model was determined using the root mean square error of calibration (RMSEC) and the root mean square error of cross-validation (RMSECV) [13]. The numbers of samples for each of the previously described calibration and validation subsets used for chemical components and TEY determinations were further reduced by the removal of sample outliers, as described by Xu et al. [26].

**Fig. 6:**
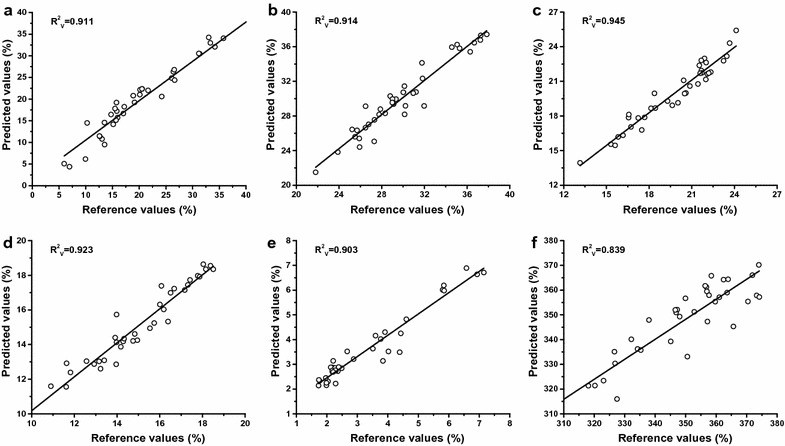
Plots of predicted versus reference values of chemical components and theoretical ethanol yield. Soluble sugar (**a**), cellulose (**b**), hemicellulose (**c**), lignin (**d**), ash (**e**), and theoretical ethanol yield (**f**) for the external validation subsets based on partial-optimized partial least squares calibration models. The $R_{\text{V}}^{2}$ represents the *square* of the correlation coefficients of the external validation subsets

Summary statistics of the partial-optimized PLS model are shown in Table 2. Based on the data pretreatment discussed above, a fair prediction of the soluble sugar, cellulose, hemicellulose, lignin, ash, and TEY was obtained using the strongest model, which exhibited low RMSEC (0.65–12.95) and RMSECV (0.74–14.18) relative to the other models. The coefficients of determination for calibration ($R_{\text{C}}^{2}$) and cross validation ($R_{\text{CV}}^{2}$) were each within the high ranges of 0.72–0.95 and 0.61–0.94, respectively. These results demonstrate the effect of SPXY enhancement on calibration of relevant bioenergy sorghum sample properties. In addition, a summary of published literature using the ratio of performance to deviation (RPD) suggested that excellent calibration models must exhibit a RPD value greater than 3, while RPD values between 2.4 and 1.5 are considered acceptable [4, 24]. Moreover, the American Association of Cereal Chemists Method 39-00 demonstrated that any model that has a range error ratio (RER) ≥10 is acceptable for quality control; if the RER of a model is greater than 15, the model is considered very good for research quantification [24]. In the present study, almost all RPD and RER values were greater than 3 and 15, respectively (except for the RER of ash) (Table 2). As a consequence, Fig. 6 illustrates that good coefficients were obtained using the square of the correlations between predicted and reference values for soluble sugar, cellulose, hemicellulose, lignin, ash, and TEY after external validation ($R_{\text{V}}^{2}$  > 0.839). All validation subsets were well predicted using the model with a relatively low root mean square error of prediction (RMSEP), which further demonstrates the utility of sample subsets optimization for prediction of multispecies feedstock properties. For the predictions for sorghum samples, partial-optimized PLS models were better than previous models for soluble sugar predication [6, 26, 40] and were superior to a series of studies that focused on cell wall components [4, 25, 26, 41] and TEY prediction [4]. In addition, a review of NIRS research published in recent years suggested that in most cases, partial-optimized PLS models are also effective for studying cellulose (glucan), hemicellulose (mainly xylan), lignin, and ash from multispecies feedstock when comparing values of $R_{\text{V}}^{2}$, RMSEP, RPD, and RER [5, 13, 16, 23, 24, 39, 42]. Therefore, past results coupled with the results of this study collectively indicate that sample subsets optimization can enhance the accuracy and robustness of NIRS quantitative analysis models for study of multispecies biomass resource streams.

**Table 2:** Summary statistics of partial-optimized partial least squares models for chemical components and theoretical ethanol yield (TEY) of bioenergy sorghum

Parameter	Calibration				Cross validation		External validation				
	Number	PCs	RMSEC	$R_{C}^{2}$	RMSECV	$R_{CV}^{2}$	Number	RMSEP	$R_{V}^{2}$	RPD	RER
Soluble sugar	108	7	2.27	0.95	2.66	0.93	37	2.57	0.91	3.39	16.42
Cellulose	108	9	0.93	0.95	1.26	0.91	37	1.18	0.91	3.46	16.30
Hemicellulose	108	7	0.75	0.95	0.85	0.94	37	0.64	0.95	4.33	25.15
Lignin	107	8	0.65	0.95	0.74	0.94	37	0.65	0.92	3.66	19.72
Ash	108	7	0.68	0.72	0.82	0.61	37	0.49	0.90	3.25	12.58
TEY	108	6	12.95	0.76	14.18	0.72	37	8.08	0.84	3.42	17.61

*PCs* principal components

### Optimization of the variable selection by CARS, SR, VIP, MC-UVE, and UVE

Due to its potential for extracting chemical information from overdetermined systems, multivariate calibration has been extensively used for applications in the analysis of multi-component spectroscopic data, especially in the NIRS field [33]. Additionally, recent approaches have shown that proper spectral variable selection has proved to be a critical step for multivariate spectroscopic calibration; many strategies have been developed to address this problem from an optimization perspective in recent years [29, 37, 43]. Competitive adaptive reweighted sampling (CARS) was proposed in 2009 by Li et al. [33]. This specialized strategy for variable selection allows for the selection of an optimal variable subset existing in the full spectra coupled with PLS regression using the simple but effective principle “survival of the fittest,” upon which Darwin’s Evolution Theory is based [33]. The selectivity ratio (SR) method, proposed first for biomarker discovery, is obtained by calculating the ratio of explained to residual variance of the *X* variables upon the *Y* target-projected component [34]. The variable importance for projection (VIP) method selected those *X* variables that contribute most to the underlying variation in the *X* variables [35]. Currently, one of the most general variable selection methods is the uninformative variable elimination (UVE) method, which evaluates the reliability of each variable in the model using stability criteria and eliminates uninformative variables. Moreover, Cai et al. [44] in 2008 reported a modified method combining UVE with Monte Carlo principles (MC-UVE) to achieve satisfactory prediction results in comparison to many other methods of wavelengths selection (MC-UVE). Although the methods listed above have been applied widely in analytical chemistry, none has yet been used to optimize the multivariate calibration model of lignocellulosic components analysis, especially for bioenergy sorghum samples. Therefore, it would be worthwhile to apply variable selection to biomass multivariate calibration modeling and conduct a comparison of five standard methods for NIRS model optimization.

**Fig. 7:**
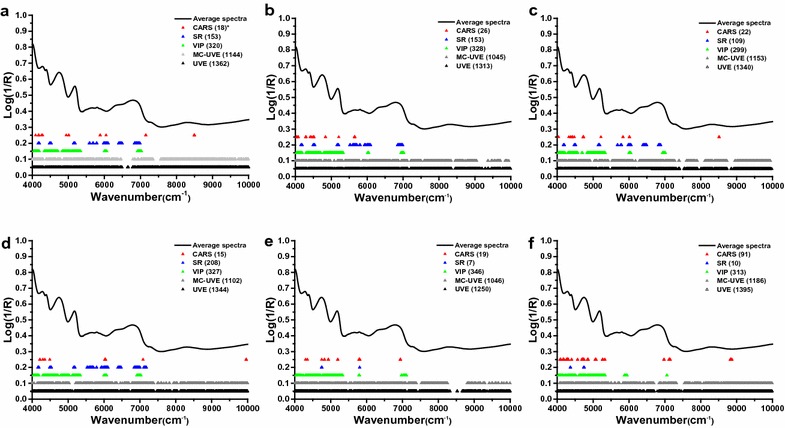
The optimal variable selection in calibration subset by CARS, SR, VIP, MC-UVE, and UVE. *CARS* competitive adaptive reweighted sampling, *SR* selectivity ratio, *VIP* variable importance for projection, *UVE* uninformative variable elimination, and *MC-UVE* UVE couple with the principle of Monte Carlo. Soluble sugar (**a**), cellulose (**b**), hemicellulose (**c**), lignin (**d**), ash (**e**), and theoretical ethanol yield (**f**). *Asterisk* refers to the number of variables selected by each method

As shown in Fig. 7, the optimal variable subsets of soluble sugar, cellulose, hemicellulose, lignin, ash, and TEY were selected using algorithms mentioned above. First, it was possible to demonstrate that the variable numbers selected by each method could be ranked in the order CARS < SR < VIP < MU-UVE < UVE for soluble sugar, cellulose, hemicellulose, and lignin (Fig. 7a–d). Meanwhile, the order of variable numbers for both ash and TEY was SR < CARS < VIP < MU-UVE < UVE (Fig. 7e, f). As a result, CARS and SR exhibited higher efficiency, as measured using “informative variables” selection, than did the other algorithms. Additionally, it could be observed that the spectral range of about 4000–7400 cm^−1^ was most effective for variable selection, consistent with our previous finding showing that to be a strong absorbance region (Figs. 3a, 7).

In order to perform a thorough analysis, we designated the characteristic variables that were selected using 5, 4, 3, 2, 1, or 0 methods as follows: “most important,” “very important,” “important,” “normal,” “less important,” and “uninformative” variables, respectively. Summary statistics for the characteristic variables of soluble sugar, cellulose, hemicellulose, lignin, ash, and TEY are provided in Table 3. Due to the basic principles that generally apply to characteristic variables, “most important” variables and “very important” variables could be considered to be the most important informative variables within the full spectra. In the present study, organic properties (soluble sugar, cellulose, hemicellulose, lignin, and TEY) achieved large numbers of “most important” variable and “very important” variable, ranging from 4 to 7 and from 37 to 64, respectively. By contrast, the number of them from ash were much lower than those of the other five properties. This might be due to the fact that ash, an inorganic mixture, is not easily or directly measurable by NIRS, as are organic properties [13, 39]. As previous studies have confirmed, the stability and accuracy of the multivariate calibration model relied heavily on strong informative variables [28]. Although the “most important” variable and “very important” variable make up only a small proportion of the full spectra, numbers of these variables unquestionably could affect the predictive performance of each property for multivariate calibration. On the other hand, judicious elimination of uninformative variables can also bolster the effectiveness of the calibration model and improve its predictive performance [45]. In this study, the “less important” variable and “uninformative” variable numbers for each property ranged from 166 to 248 and from 132 to 269, accounting for 10.7–15.9% and 8.5–17.3% of the total 1557 variables observed, respectively (Table 3). Therefore, variable selection, an effective optimization strategy, could be an indispensable step for NIRS modeling of lignocellulosic feedstock. Furthermore, the five optimal variable subsets selected here provide very useful information to facilitate further biomass components prediction (Fig. 7).

**Table 3:** The statistics of characteristic variables for chemical components and theoretical ethanol yield (TEY)

Parameter	MIV		VIV		IV		NV		LIV		UIV	
	*N*	*P* (%)	*N*	*P* (%)	*N*	*P* (%)	*N*	*P* (%)	*N*	*P* (%)	*N*	*P* (%)
Soluble sugar	4	0.3	64	4.1	230	14.8	908	58.3	215	13.8	136	8.7
Cellulose	5	0.3	56	3.6	240	15.4	824	52.9	248	15.9	184	11.8
Hemicellulose	4	0.3	37	2.4	251	16.1	918	59.0	166	10.7	181	11.6
Lignin	7	0.5	62	4.0	276	17.7	826	53.1	233	15.0	153	9.8
Ash	1	0.1	14	1.0	269	17.3	796	51.1	208	13.4	269	17.3
TEY	4	0.3	55	3.5	234	15.0	921	59.2	211	13.6	132	8.5

*MIV* most important variable, *VIV* very important variable, *IV* important variable, *NV* normal variable, *LIV* less important variable, *UIV* uninformative variable, *N* number of selected variables, *P* the proportion of selected variables in total variables

### Dual-optimized PLS models

**Fig. 8:**
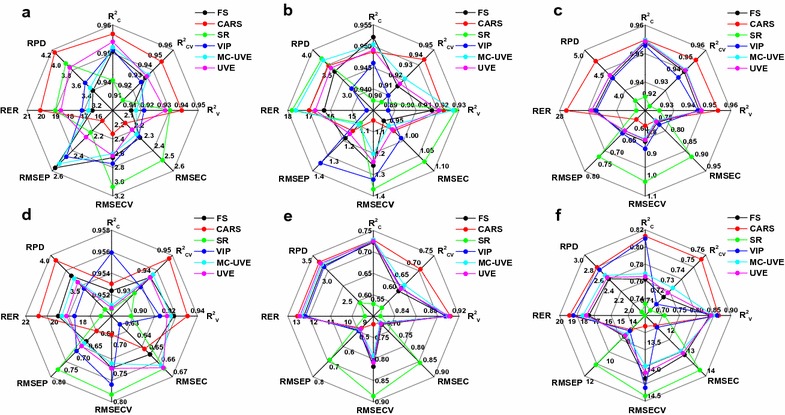
The performance of 30 dual-optimized PLS models and 6 partial-optimized PLS models. *FS* full spectra, *CARS* competitive adaptive reweighted sampling, *SR* selectivity ratio, *VIP* variable importance for projection, *UVE* uninformative variable elimination, *MC-UVE* UVE couple with the principle of Monte Carlo. Soluble sugar (**a**), cellulose (**b**), hemicellulose (**c**), lignin (**d**), ash (**e**), theoretical ethanol yield (**f**)

Coupled with SPXY-optimized sample subsets, five methods for variable selection were employed to develop 30 dual-optimized PLS models for quantitative analysis of soluble sugar, cellulose, hemicellulose, lignin, ash, and TEY. Meanwhile, the six partial-optimized PLS models described above served as controls. Summary statistics for a total of 36 defined models are presented in Table 2 and Additional file 1: Tables A3–A7. Compared with other dual-optimized models, CARS-SPXY exhibited the greatest positive impact upon the stability of PLS models when comparing values of principal components (5–8), $R_{\text{C}}^{2}$ (0.73–0.96), RMSEC (0.65–11.50), $R_{\text{CV}}^{2}$ (0.69–0.95), and RMSECV (0.68–13.20) (Fig. 8 and Additional file 1: Table A3). Furthermore, lower RMSEP (0.48–7.41) and higher RPD (3.34–4.73) and RER (12.94–27.45) values were obtained using the robust CARS-SPXY dual-optimized PLS models, which were significantly better than those of four other dual-optimized models for the most relevant properties. Notably, the use of one in four samples for external validation led to strong correlations between predicted and reference values (Additional file 2: Fig. A1). These results demonstrate that CARS-SPXY was the best method to clearly improve upon the accuracies of the prediction performance of the PLS model among these five dual-optimized methods. These results are consistent with our previously stated results that CARS displayed relatively higher efficiency over other methods in the selection of informative variables as well as for judicious elimination of uninformative variables (Fig. 7).

Meanwhile, the sample subsets and spectral variable subsets were optimized using VIP-SPXY, MC-UVE-SPXY, and UVE-SPXY models using the following parameters: a low number of principal components; high $R_{\text{C}}^{2}$, $R_{\text{CV}}^{2}$, $R_{\text{V}}^{2}$; low RMSEC, RMSECV, RMSEP; and high RPD, RER (Additional file 1: Tables A5–A7). Additional file 2: Figures A3–A5 demonstrate that all relevant properties were successfully predicted by the three models mentioned above, which generally displayed relatively higher correlations between predicted and reference values than controls (Fig. 6). These results indicated that VIP-SPXY, MC-UVE-SPXY, and UVE-SPXY models could achieve higher performance for predicting soluble sugar, cellulose, hemicellulose, lignin, ash, and TEY (Fig. 8). Conversely, it was found that SR-SPXY significantly decreased the stability of the calibration models because the RMSEC and RMSECV for each property sharply increased. Meanwhile, significant decreases in $R_{\text{C}}^{2}$ and $R_{\text{CV}}^{2}$ were observed in the present study which were generally lower than controls (Table 2; Additional file 1: Table A4). As a consequence, RPD and RER decreased significantly and relatively lower $R_{\text{V}}^{2}$ values were obtained for the validation set of each relevant property. These results probably stem from the inability of the SR method to achieve informative variable selection and uninformative variables elimination (Figs. 7, 8; Additional file 1: Table A4 and Additional file 2: Figure A2).

In summary, dual-optimized PLS models generally display significantly higher robustness and accuracy than partial-optimized PLS models for the prediction of relevant properties by utilizing dual optimization of sample subset partitioning and variable selection. Additionally, CARS-SPXY was the most effective dual-optimization method for improving the predictive performance of the PLS model in this study (Fig. 8).

## Conclusions

A large number of bioenergy sorghum samples, including 70 sweet sorghum and 77 biomass sorghum samples, exhibited diverse chemical components and high potential for bioethanol production. Using the NIRS of total 147 samples, a qualitative analysis between sweet sorghum and biomass sorghum was conducted via the linear discriminant analysis model and 99.3% of samples were correctly classified using a set of 20 principal components. Meanwhile, sample subset partitioning and variable selection could substantially enhance the predictive performance of a PLS model for determination of chemical components and TEY. These dual-optimized models generally achieved high $R_{\text{C}}^{2}$, $R_{\text{CV}}^{2}$, $R_{\text{V}}^{2}$, RPD, and RER values and low RESEC, RESECV, and RMSEP values relative to partial-optimized models, demonstrating relatively higher enhancement of both robustness and accuracy of prediction models. Finally, comparative analyses of five dual-optimized methods indicate that CARS-SPXY was the most efficient method for improving the predictive performance of PLS multivariate calibration. Therefore, high-throughput dual-optimized NIRS models could facilitate feedstock selection, bioenergy crop breeding, and genetic modification for efficient biofuel production.

## Methods

### Sample selection and preparation

To select bioenergy sorghum samples with diverse genetic, environment, and cultivation background is of importance for accuracy and robustness model building. Since 2006, National Energy R&D Center for Non-food Biomass (NECB) has collected nationwide sorghum germplasm resources to make hybrid breeding for a bioethanol purpose. Till 2013, the most representative six sweet type and six biomass type hybrids have been developed by NECB for commercial scale production. To evaluate the yield performance and energy potential, we planted these twelve bioenergy sorghum varieties under different cultivation treatments including plant spacing and fertilizer rate, in Zhuozhou, Hebei and Uxin, Inner Mongolia in 2013 and 2014. The two study sites were some 800 km apart in northern China. A total of 147 representative samples were taken at the two sites for the high-throughput NIRS models building in this study.

All sorghum samples were harvested on their dates of physiological maturity after a growth period of between 110 and 150 days. Preparation of samples was performed according to the process described by Li et al. [3] with minor modifications. In this study, stem samples were ground using a crusher mill into particles 1–2 cm in size. The particles were first dried at 45 °C for 48 h after inactivation at 105 °C for 20 min. Next, the dried particles were ground into powders and passed through a combined −40/+80 mesh screen. A total of 147 mesh-screened samples (as dry matter) were stored in a dry container until use.

### Biomass compositional analysis and TEY calculation

The soluble sugar was extracted with distilled water and determined by the anthrone/H_2_SO_4_ method using a UV–VIS spectrometer (TU-1901, Beijing Purkinje Instruments Co. Ltd., Beijing, China) according to Li et al. [3] The standard curve was plotted using d-glucose as the standard (purchased from Xilong Scientific Co., Ltd., China).

The structural carbohydrates (i.e., glucose, xylose, and arabinose) and lignin (acid-soluble lignin and acid-insoluble lignin) were extracted using a two-step sulfuric acid hydrolysis process. Sample quantity and composition were measured using an HPLC system (1260 series, Agilent Technologies, Santa Clara, CA, USA) equipped with an Aminex HPX-87H chromatography column (300 × 7.8 mm, particle size 9 µm, Bio-Rad Laboratories, Hercules, CA, USA), a UV–VIS spectrometer (TU-1901, Beijing Purkinje Instruments Co. Ltd.) and a muffle furnace (VULCAN 3-550, Densply International Inc., York, PA, USA) according to the Laboratory Analytical Procedures from National Renewable Energy Laboratory [46]. Dry matter (2 g per sample) was added to ceramic crucibles (30 mL volume) to determine ash content after incineration in the muffle furnace. Cellulose content was calculated from the glucose content. Hemicellulose was calculated from the sum of xylose and arabinose contents. Lignin was calculated from the sum of acid-soluble lignin and acid-insoluble lignin contents.

Theoretical ethanol yield (TEY), as reported by Zhao et al. [47], was calculated from the C6 sugar (soluble sugar and cellulose), C5 sugar (hemicellulose), and total sugar (C6 sugar and C5 sugar) in the dry matter, with minor modifications. In order to obtain the TEY results in g/kg, the equation below was modified and the term dry biomass (t/ha) was removed.

$$\begin{aligned} {\text{TEY-C6 }}\left( {{\text{g}}/{\text{kg}}} \right) & = \left[ {{\text{soluble sugar }}\left( \% \right) + {\text{cellulose }}\left( \% \right) \times 1.11 \,\left( {\text{conversion factor of sugar from cellulose}} \right)} \right] \\ & \quad \times\, 0. 5 1 { }\left( {\text{conversion factor of ethanol from sugar}} \right) \\ & \quad \times\, 0. 8 5 { }\left( {\text{process efficiency of ethanol from sugar}} \right) \\ & \quad \times\, 1000/0.79 { }\left( {{\text{specific gravity of ethanol}},{\text{ g}}\,{\text{mL}}^{ - 1} } \right) \hfill \\ \end{aligned}$$

$$\begin{aligned} {\text{TEY-C5 }}\left( {{\text{g}}/{\text{kg}}} \right) & = {\text{hemicellulose}}\left( \% \right) \times 1.11 \, \left( {\text{conversion factor of sugar from hemicellulose}} \right) \\ & \quad \times\,0. 5 1 { }\left( {\text{conversion factor of ethanol from sugar}} \right) \\ & \quad \times\, 0. 8 5 { }\left( {\text{process efficiency of ethanol from sugar}} \right) \\ & \quad \times\, 1000/0. 7 9 { }\left( {{\text{specific gravity of ethanol}},{\text{ g}}\,{\text{mL}}^{ - 1} } \right) \\ \end{aligned}$$

$${\text{TEY}} ({\text{g}}/{\text{kg}})={\text{TEY-C6}}\,({\text{g}}/{\text{kg}})+{\text{TEY-C5}}\,({\text{g}}/{\text{kg}})$$

The results of TEY values could be found in Additional file 1: Table A2.

### NIRS measurement and pretreatments

All dry matter was scanned and recorded from three separate samplings using a Thermo Antaris II FT-NIR (Thermo Scientific Inc., Madison, WI, USA) equipped with a diffuse reflectance accessory. Each spectrum was averaged from 64 scans at a resolution of 8 cm^−1^ in the wavenumber range of 4000–10,000 cm^−1^, including 1557 spectral variables. The spectrometer was controlled and data were acquired using TQ Analyst software (ver. 9.3.107, Thermo Scientific Inc.). Spectra were first adjusted using multiplicative scatter correction to correct spectra for scatter. Next, a Norris derivative filter was used to reduce random noise. The first derivative was used to resolve spectra peak overlap and eliminate linear baseline drift [26]. The purpose of the corrections above was to remove multiplicative and additive effects stemming from instrument settings or variations caused by sample and environmental conditions [25].

### Qualitative analysis

A principal component analysis model was calculated using TQ Analyst 9.3 to evaluate the spectral distribution of bioenergy sorghum samples based on 3D plots (Fig. 3b). The principal component analysis models included 147 samples and incorporated a maximum number of 40 principal components. As a well-known and frequently used technique, linear discriminant analysis was employed for data classification in this study. The Mahalanobis distance was determined between the samples and each sorghum class center. Meanwhile, the correct classification rate was calculated to obtain a classification result according to He et al. [15].

### Quantitative analysis

In the present study, a stepwise procedure reported by Galvão et al. [32] was employed to select samples according to their differences in both measured properties and NIRS. Based on this method, one of every four samples was sorted into external validation subsets using ChemDataSolution, ver. 2.0 (Dalian ChemData Solution Technology Co. Ltd., Dalian, China) to obtain a fair multivariate prediction [48] and the remaining samples were used for the calibration subsets.

The previously described NIRS variables were selected using the software ChemDataSolution ver. 2.0. Before determination of quantitative chemical components and TEY, CARS, SR, VIP, MC-UVE, and UVE models were employed for NIRS optimization in this study.

A total of 36 PLS multivariate calibrations were developed using TQ Analyst software (ver. 9.3.107) [49], which predicted one property at a time based on partial optimization or dual optimization in the present study. The “leave-one-out” method was recommended for cross-validation when developing PLS models, in order to select the optimal number of factors and to avoid over-fitting [13, 25]. The performance of multivariate calibrations was evaluated using RMSEC, RMSECV, RMSEP, and $R_{\text{C}}^{2}$, $R_{\text{CV}}^{2}$ , and $R_{\text{V}}^{2}$. Furthermore, the RPD and the RER were also calculated to ascertain the potential of the NIRS models for application to breeding or industry screening [1, 24, 25, 50].

### Statistical analysis

All chemical assays were conducted in triplicate and the average values are presented as percentage of dry weight. Correlation coefficients were calculated by performing Pearson's rank correlation analysis using IBM SPSS Statistics V.22. This analysis used mean values and coefficient coefficients of variation were calculated from all original determinations and defined as the ratio of the standard deviation to the mean value.

## Additional files

**Additional file 1: Table A1.** Descriptive statistics of chemical components (%) for the 147 bioenergy sorghum samples. **Table A2.** Descriptive statistics of theoretical ethanol yield (TEY, g/kg) for the 147 bioenergy sorghum samples. **Table A3.** Summary statistics of CARS-SPXY dual optimized PLS model for the determination of soluble sugar, cellulose, hemicellulose, lignin and theoretical ethanol yield (TEY). **Table A4.** Summary statistics of SR-SPXY dual optimized PLS model for the determination of soluble sugar, cellulose, hemicellulose, lignin and theoretical ethanol yield (TEY). **Table A5.** Summary statistics of VIP-SPXY dual optimized PLS model for the determination of soluble sugar, cellulose, hemicellulose, lignin and theoretical ethanol yield (TEY). **Table A6.** Summary statistics of MC-UVE-SPXY dual optimized PLS model for the determination of soluble sugar, cellulose, hemicellulose, lignin and theoretical ethanol yield (TEY). **Table A7.** Summary statistics of UVE-SPXY dual optimized PLS model for the determination of soluble sugar, cellulose, hemicellulose, lignin and theoretical ethanol yield (TEY).

**Additional file 2: Figure A1.** Plots of predicted versus measured values of parameters. Soluble sugar (a), cellulose (b), hemicellulose (c), lignin (d), ash (e), and theoretical ethanol yield (f) for the external validation subsets based on CARS-SPXY dual-optimized PLS models. The ${\text{R}}_{\text{V}}^{2}$ represents the square of the correlation coefficients of the external validation subsets. **Figure A2.** Plots of predicted versus measured value of parameters. Soluble sugar (a), cellulose (b), hemicellulose (c), lignin (d), ash (e), and theoretical ethanol yield (f) for the external validation subsets based on SR-SPXY dual-optimized PLS models. The ${\text{R}}_{\text{V}}^{2}$ represents the square of the correlation coefficients of the external validation subsets. **Figure A3.** Plots of predicted versus measured value of parameters. Soluble sugar (a), cellulose (b), hemicellulose (c), lignin (d), ash (e), and theoretical ethanol yield (f) for the external validation subsets based on VIP-SPXY dual-optimized PLS models. The ${\text{R}}_{\text{V}}^{2}$ represents the square of the correlation coefficients of the external validation subsets. **Figure A4.** Plots of predicted versus measured value of parameters. Soluble sugar (a), cellulose (b), hemicellulose (c), lignin (d), ash (e), and theoretical ethanol yield (f) for the external validation subsets based on MC-UVE-SPXY dual-optimized PLS models. The ${\text{R}}_{\text{V}}^{2}$ represents the square of the correlation coefficients of the external validation subsets. **Figure A5.** Plots of predicted versus measured value of parameters. Soluble sugar (a), cellulose (b), hemicellulose (c), lignin (d), ash (e), and theoretical ethanol yield (f) for the external validation subsets based on UVE-SPXY dual-optimized PLS models. The ${\text{R}}_{\text{V}}^{2}$ represents the square of the correlation coefficients of the external validation subsets.

## Abbreviations

TEY: theoretical ethanol yield
NIRS: near infrared spectroscopy
SPXY: sample set partitioning based on joint *X*–*Y* distances method
PLS: partial least squares
CARS: competitive adaptive reweighted sampling
SR: selectivity ratio
VIP: variable importance for projection
UVE: uninformative variable elimination
MC-UVE: UVE couple with the principle of Monte Carlo
