# Supplementary material for: High-throughput analysis of chemical components and theoretical ethanol yield of dedicated bioenergy sorghum using dual-optimized partial least squares calibration models
Source: Biotechnol Biofuels. 2017 Sep 4;10:206. doi: 10.1186/s13068-017-0892-z (PMC5584014; doi:10.1186/s13068-017-0892-z)
Supplement: Supplementary file 1 — Additional file 1: Table A1. Descriptive statistics of chemical components (%) for the 147 bioenergy sorghum samples. Table A2. Descriptive statistics of theoretical ethanol yield (TEY, g/kg) for the 147 bioenergy sorghum samples. Table A3. Summary statistics of CARS-SPXY dual optimized PLS model for the determination of soluble sugar, cellulose, hemicellulose, lignin and theoretical ethanol yield (TEY). Table A4. Summary statistics of SR-SPXY dual optimized PLS model for the determination of soluble sugar, cellulose, hemicellulose, lignin and theoretical ethanol yield (TEY). Table A5. Summary statistics of VIP-SPXY dual optimized PLS model for the determination of soluble sugar, cellulose, hemicellulose, lignin and theoretical ethanol yield (TEY). Table A6. Summary statistics of MC-UVE-SPXY dual optimized PLS model for the determination of soluble sugar, cellulose, hemicellulose, lignin and theoretical ethanol yield (TEY). Table A7. Summary statistics of UVE-SPXY dual optimized PLS model for the determination of soluble sugar, cellulose, hemicellulose, lignin and theoretical ethanol yield (TEY). [file 13068_2017_892_MOESM1_ESM.pptx]

## Slide 1
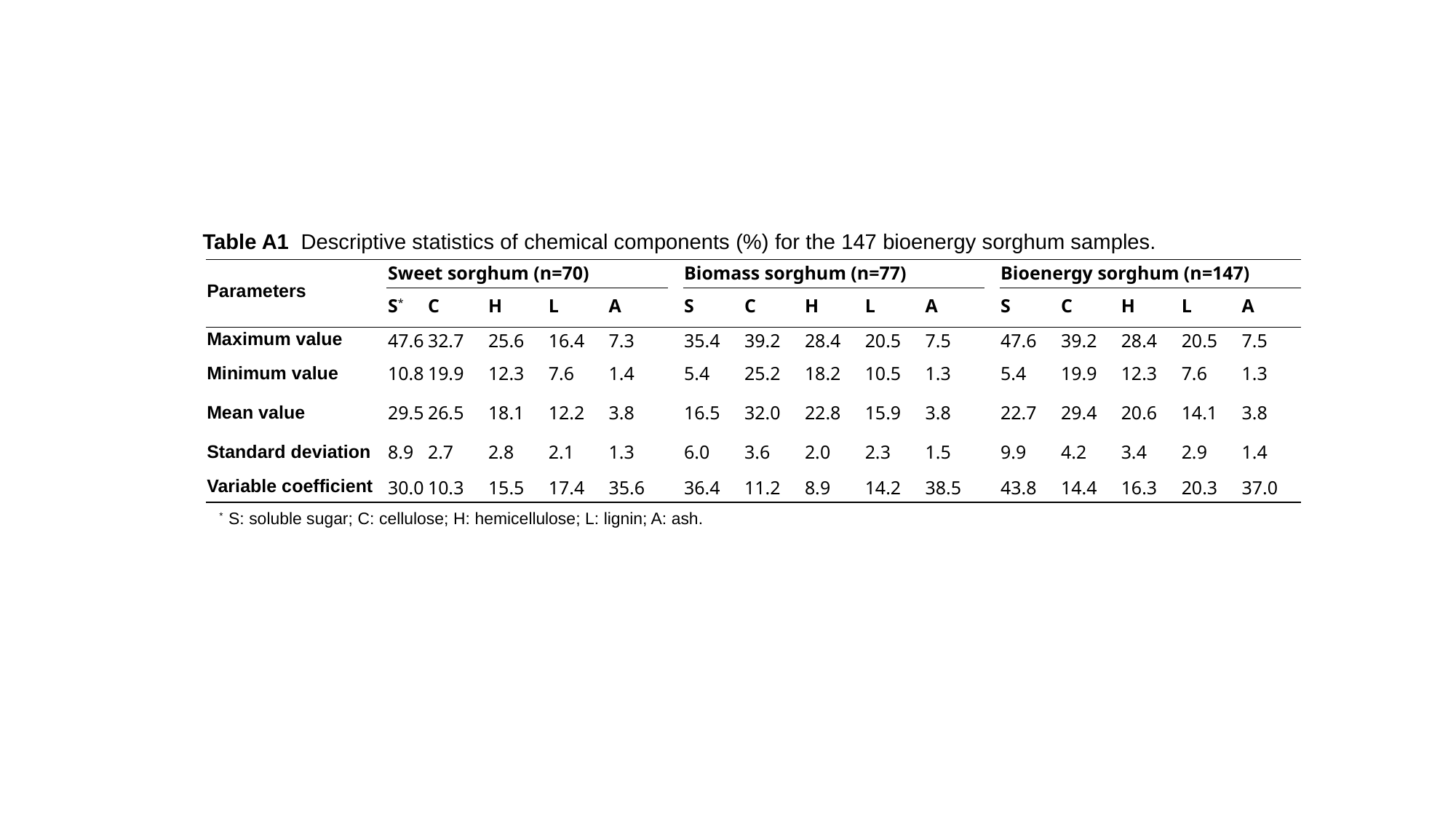

Table A1 Descriptive statistics of chemical components (%) for the 147 bioenergy sorghum samples.
| Parameters | Sweet sorghum (n=70) | | | | | | Biomass sorghum (n=77) | | | | | | Bioenergy sorghum (n=147) | | | | |
| --- | --- | --- | --- | --- | --- | --- | --- | --- | --- | --- | --- | --- | --- | --- | --- | --- | --- |
| | S\* | C | H | L | A | | S | C | H | L | A | | S | C | H | L | A |
| Maximum value | 47.6 | 32.7 | 25.6 | 16.4 | 7.3 | | 35.4 | 39.2 | 28.4 | 20.5 | 7.5 | | 47.6 | 39.2 | 28.4 | 20.5 | 7.5 |
| Minimum value | 10.8 | 19.9 | 12.3 | 7.6 | 1.4 | | 5.4 | 25.2 | 18.2 | 10.5 | 1.3 | | 5.4 | 19.9 | 12.3 | 7.6 | 1.3 |
| Mean value | 29.5 | 26.5 | 18.1 | 12.2 | 3.8 | | 16.5 | 32.0 | 22.8 | 15.9 | 3.8 | | 22.7 | 29.4 | 20.6 | 14.1 | 3.8 |
| Standard deviation | 8.9 | 2.7 | 2.8 | 2.1 | 1.3 | | 6.0 | 3.6 | 2.0 | 2.3 | 1.5 | | 9.9 | 4.2 | 3.4 | 2.9 | 1.4 |
| Variable coefficient | 30.0 | 10.3 | 15.5 | 17.4 | 35.6 | | 36.4 | 11.2 | 8.9 | 14.2 | 38.5 | | 43.8 | 14.4 | 16.3 | 20.3 | 37.0 |
* S: soluble sugar; C: cellulose; H: hemicellulose; L: lignin; A: ash.

## Slide 2
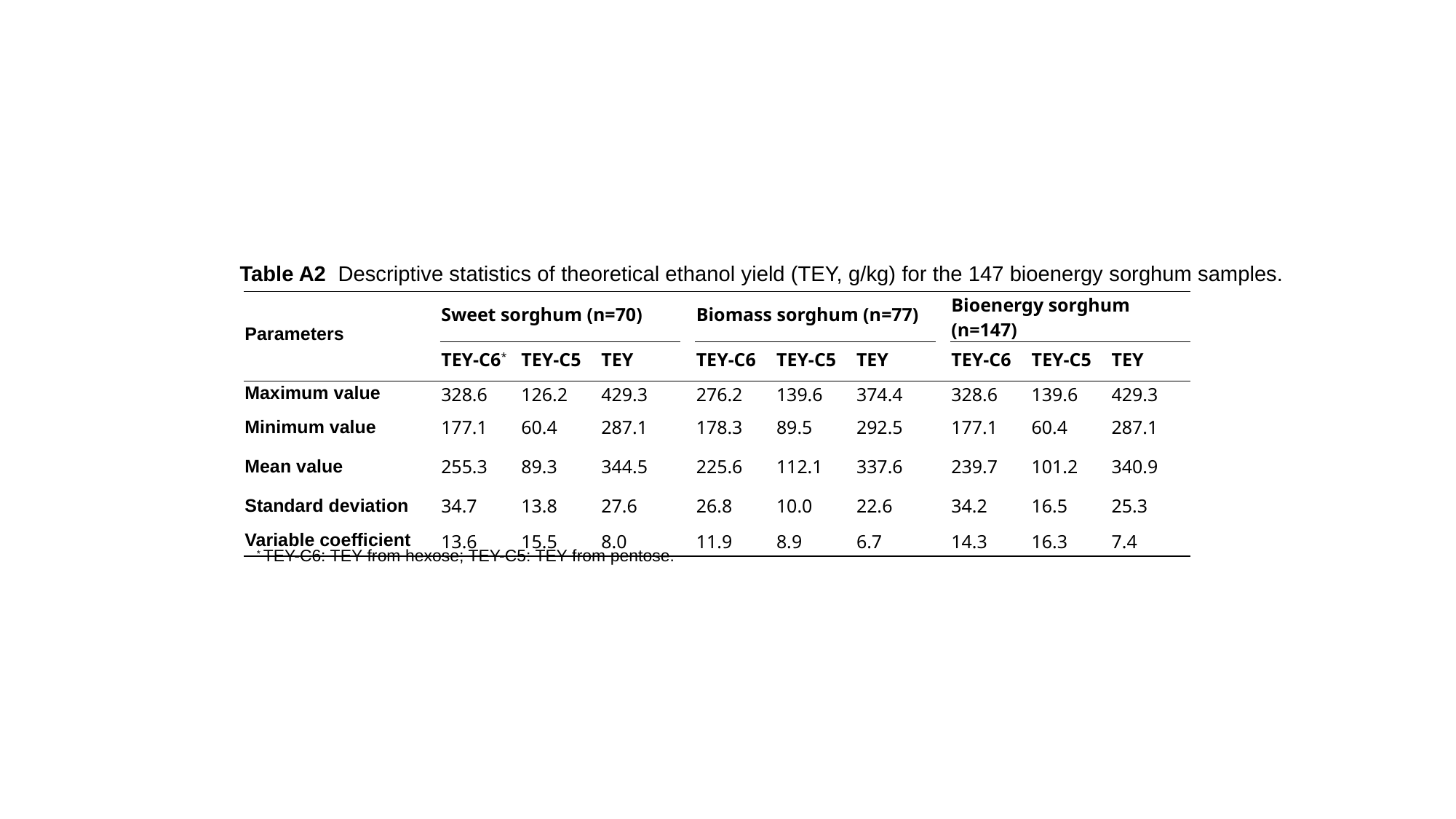

Table A2 Descriptive statistics of theoretical ethanol yield (TEY, g/kg) for the 147 bioenergy sorghum samples.
| Parameters | Sweet sorghum (n=70) | | | | Biomass sorghum (n=77) | | | | Bioenergy sorghum (n=147) | | |
| --- | --- | --- | --- | --- | --- | --- | --- | --- | --- | --- | --- |
| | TEY-C6\* | TEY-C5 | TEY | | TEY-C6 | TEY-C5 | TEY | | TEY-C6 | TEY-C5 | TEY |
| Maximum value | 328.6 | 126.2 | 429.3 | | 276.2 | 139.6 | 374.4 | | 328.6 | 139.6 | 429.3 |
| Minimum value | 177.1 | 60.4 | 287.1 | | 178.3 | 89.5 | 292.5 | | 177.1 | 60.4 | 287.1 |
| Mean value | 255.3 | 89.3 | 344.5 | | 225.6 | 112.1 | 337.6 | | 239.7 | 101.2 | 340.9 |
| Standard deviation | 34.7 | 13.8 | 27.6 | | 26.8 | 10.0 | 22.6 | | 34.2 | 16.5 | 25.3 |
| Variable coefficient | 13.6 | 15.5 | 8.0 | | 11.9 | 8.9 | 6.7 | | 14.3 | 16.3 | 7.4 |
* TEY-C6: TEY from hexose; TEY-C5: TEY from pentose.

## Slide 3
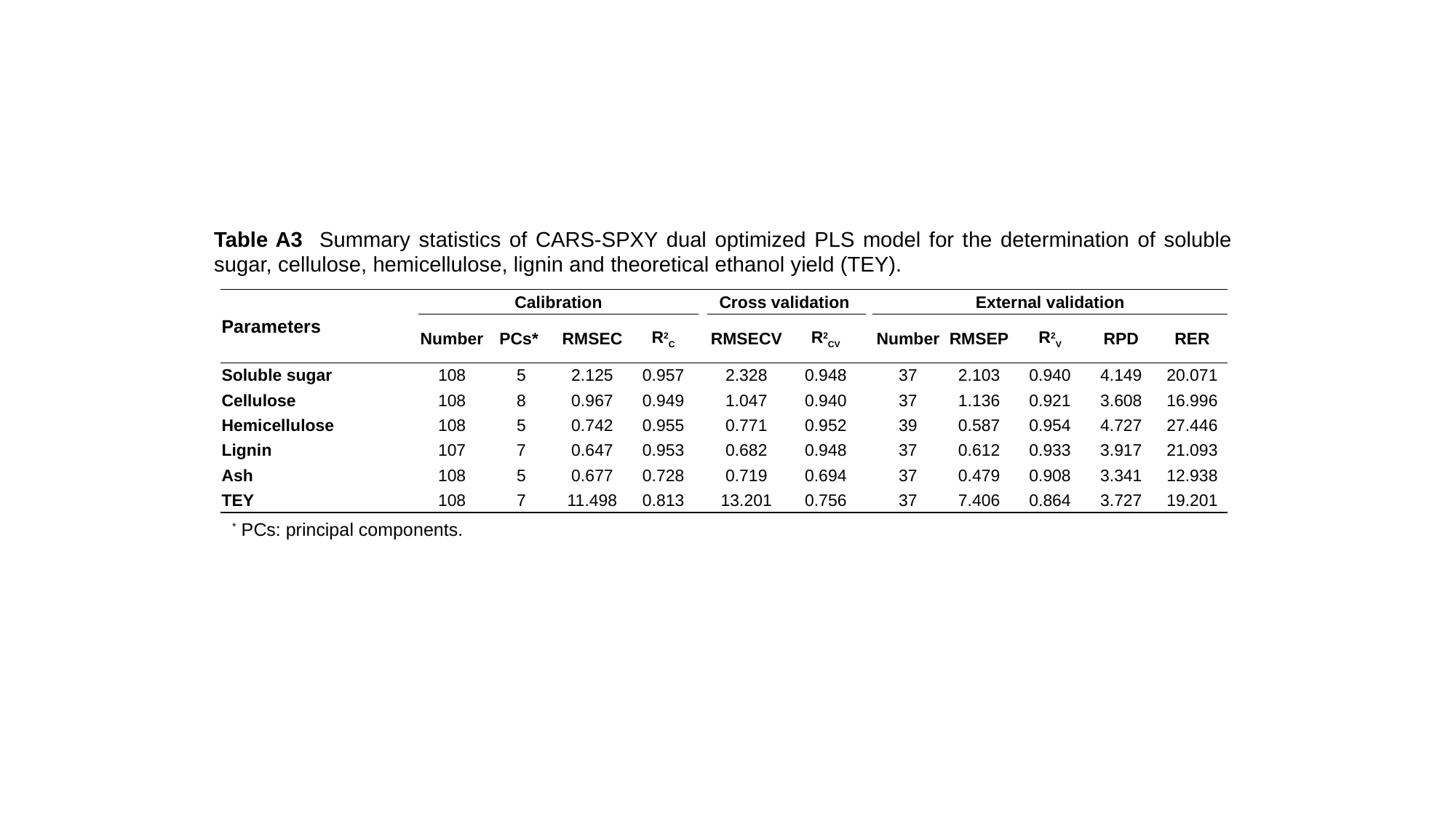

Table A3 Summary statistics of CARS-SPXY dual optimized PLS model for the determination of soluble sugar, cellulose, hemicellulose, lignin and theoretical ethanol yield (TEY).
| Parameters | Calibration | | | | | Cross validation | | | External validation | | | | |
| --- | --- | --- | --- | --- | --- | --- | --- | --- | --- | --- | --- | --- | --- |
| | Number | PCs\* | RMSEC | R2C | | RMSECV | R2CV | | Number | RMSEP | R2V | RPD | RER |
| Soluble sugar | 108 | 5 | 2.125 | 0.957 | | 2.328 | 0.948 | | 37 | 2.103 | 0.940 | 4.149 | 20.071 |
| Cellulose | 108 | 8 | 0.967 | 0.949 | | 1.047 | 0.940 | | 37 | 1.136 | 0.921 | 3.608 | 16.996 |
| Hemicellulose | 108 | 5 | 0.742 | 0.955 | | 0.771 | 0.952 | | 39 | 0.587 | 0.954 | 4.727 | 27.446 |
| Lignin | 107 | 7 | 0.647 | 0.953 | | 0.682 | 0.948 | | 37 | 0.612 | 0.933 | 3.917 | 21.093 |
| Ash | 108 | 5 | 0.677 | 0.728 | | 0.719 | 0.694 | | 37 | 0.479 | 0.908 | 3.341 | 12.938 |
| TEY | 108 | 7 | 11.498 | 0.813 | | 13.201 | 0.756 | | 37 | 7.406 | 0.864 | 3.727 | 19.201 |
* PCs: principal components.

## Slide 4
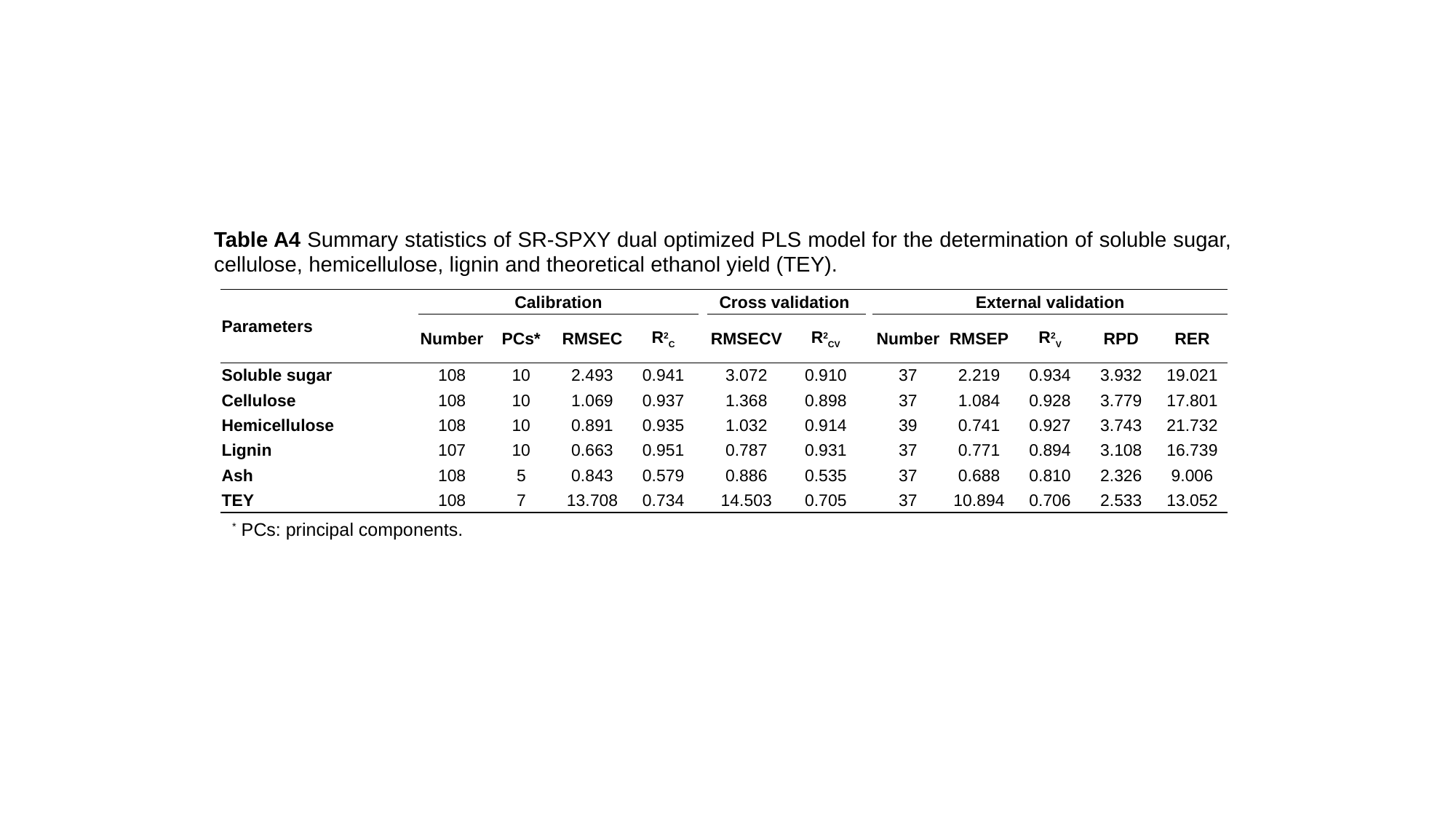

Table A4 Summary statistics of SR-SPXY dual optimized PLS model for the determination of soluble sugar, cellulose, hemicellulose, lignin and theoretical ethanol yield (TEY).
| Parameters | Calibration | | | | | Cross validation | | | External validation | | | | |
| --- | --- | --- | --- | --- | --- | --- | --- | --- | --- | --- | --- | --- | --- |
| | Number | PCs\* | RMSEC | R2C | | RMSECV | R2CV | | Number | RMSEP | R2V | RPD | RER |
| Soluble sugar | 108 | 10 | 2.493 | 0.941 | | 3.072 | 0.910 | | 37 | 2.219 | 0.934 | 3.932 | 19.021 |
| Cellulose | 108 | 10 | 1.069 | 0.937 | | 1.368 | 0.898 | | 37 | 1.084 | 0.928 | 3.779 | 17.801 |
| Hemicellulose | 108 | 10 | 0.891 | 0.935 | | 1.032 | 0.914 | | 39 | 0.741 | 0.927 | 3.743 | 21.732 |
| Lignin | 107 | 10 | 0.663 | 0.951 | | 0.787 | 0.931 | | 37 | 0.771 | 0.894 | 3.108 | 16.739 |
| Ash | 108 | 5 | 0.843 | 0.579 | | 0.886 | 0.535 | | 37 | 0.688 | 0.810 | 2.326 | 9.006 |
| TEY | 108 | 7 | 13.708 | 0.734 | | 14.503 | 0.705 | | 37 | 10.894 | 0.706 | 2.533 | 13.052 |
* PCs: principal components.

## Slide 5
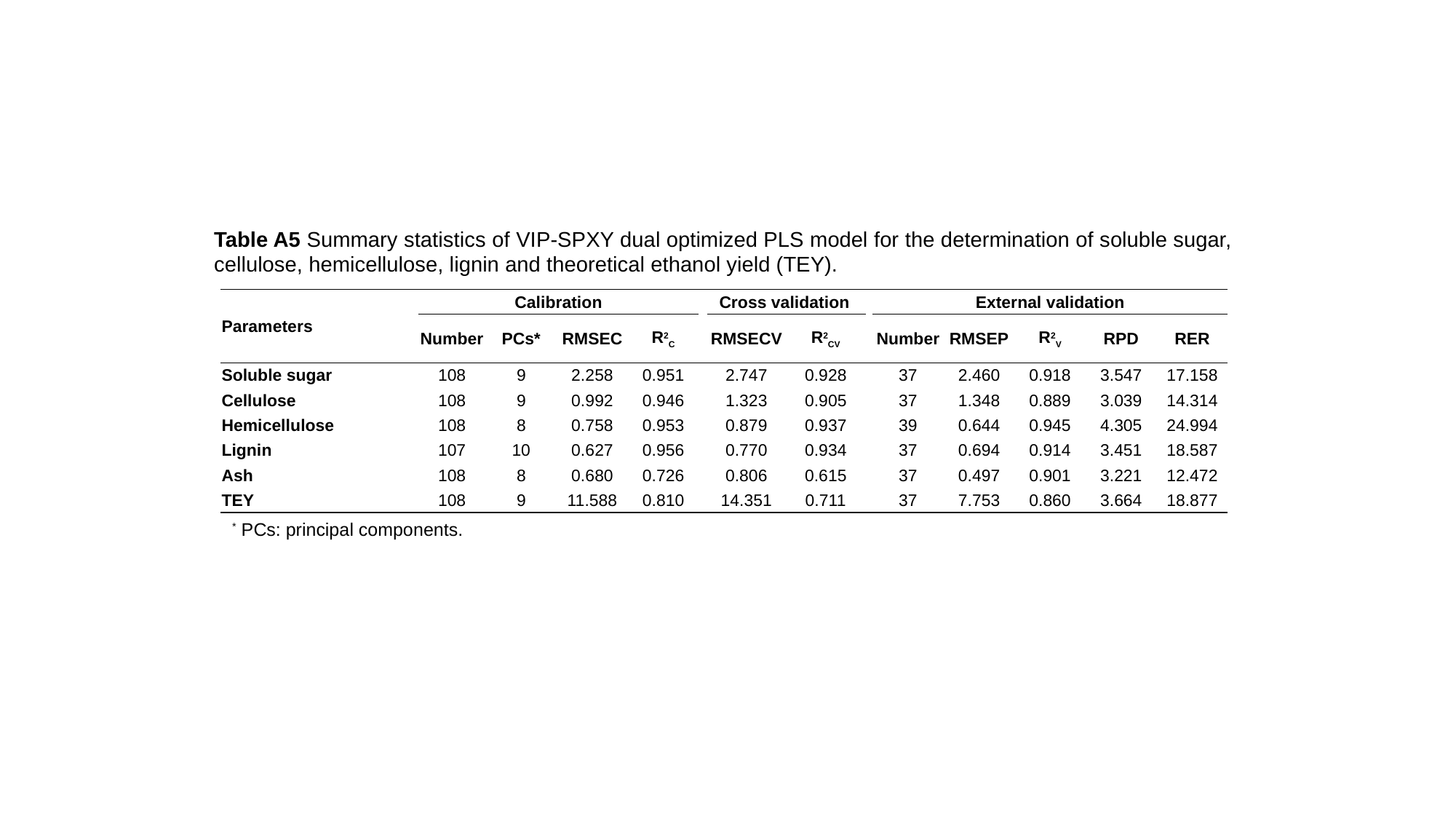

Table A5 Summary statistics of VIP-SPXY dual optimized PLS model for the determination of soluble sugar, cellulose, hemicellulose, lignin and theoretical ethanol yield (TEY).
| Parameters | Calibration | | | | | Cross validation | | | External validation | | | | |
| --- | --- | --- | --- | --- | --- | --- | --- | --- | --- | --- | --- | --- | --- |
| | Number | PCs\* | RMSEC | R2C | | RMSECV | R2CV | | Number | RMSEP | R2V | RPD | RER |
| Soluble sugar | 108 | 9 | 2.258 | 0.951 | | 2.747 | 0.928 | | 37 | 2.460 | 0.918 | 3.547 | 17.158 |
| Cellulose | 108 | 9 | 0.992 | 0.946 | | 1.323 | 0.905 | | 37 | 1.348 | 0.889 | 3.039 | 14.314 |
| Hemicellulose | 108 | 8 | 0.758 | 0.953 | | 0.879 | 0.937 | | 39 | 0.644 | 0.945 | 4.305 | 24.994 |
| Lignin | 107 | 10 | 0.627 | 0.956 | | 0.770 | 0.934 | | 37 | 0.694 | 0.914 | 3.451 | 18.587 |
| Ash | 108 | 8 | 0.680 | 0.726 | | 0.806 | 0.615 | | 37 | 0.497 | 0.901 | 3.221 | 12.472 |
| TEY | 108 | 9 | 11.588 | 0.810 | | 14.351 | 0.711 | | 37 | 7.753 | 0.860 | 3.664 | 18.877 |
* PCs: principal components.

## Slide 6
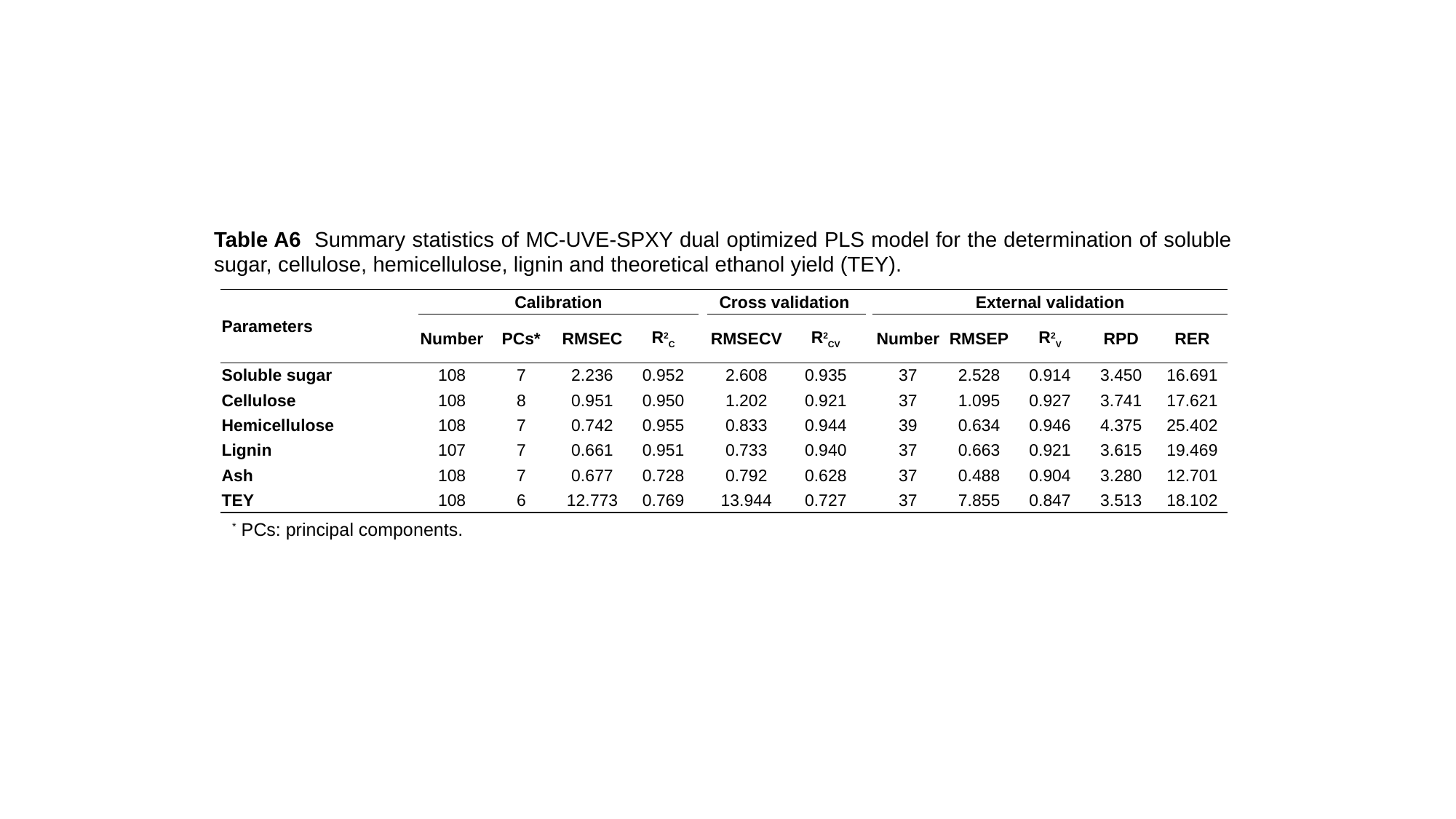

Table A6 Summary statistics of MC-UVE-SPXY dual optimized PLS model for the determination of soluble sugar, cellulose, hemicellulose, lignin and theoretical ethanol yield (TEY).
| Parameters | Calibration | | | | | Cross validation | | | External validation | | | | |
| --- | --- | --- | --- | --- | --- | --- | --- | --- | --- | --- | --- | --- | --- |
| | Number | PCs\* | RMSEC | R2C | | RMSECV | R2CV | | Number | RMSEP | R2V | RPD | RER |
| Soluble sugar | 108 | 7 | 2.236 | 0.952 | | 2.608 | 0.935 | | 37 | 2.528 | 0.914 | 3.450 | 16.691 |
| Cellulose | 108 | 8 | 0.951 | 0.950 | | 1.202 | 0.921 | | 37 | 1.095 | 0.927 | 3.741 | 17.621 |
| Hemicellulose | 108 | 7 | 0.742 | 0.955 | | 0.833 | 0.944 | | 39 | 0.634 | 0.946 | 4.375 | 25.402 |
| Lignin | 107 | 7 | 0.661 | 0.951 | | 0.733 | 0.940 | | 37 | 0.663 | 0.921 | 3.615 | 19.469 |
| Ash | 108 | 7 | 0.677 | 0.728 | | 0.792 | 0.628 | | 37 | 0.488 | 0.904 | 3.280 | 12.701 |
| TEY | 108 | 6 | 12.773 | 0.769 | | 13.944 | 0.727 | | 37 | 7.855 | 0.847 | 3.513 | 18.102 |
* PCs: principal components.

## Slide 7
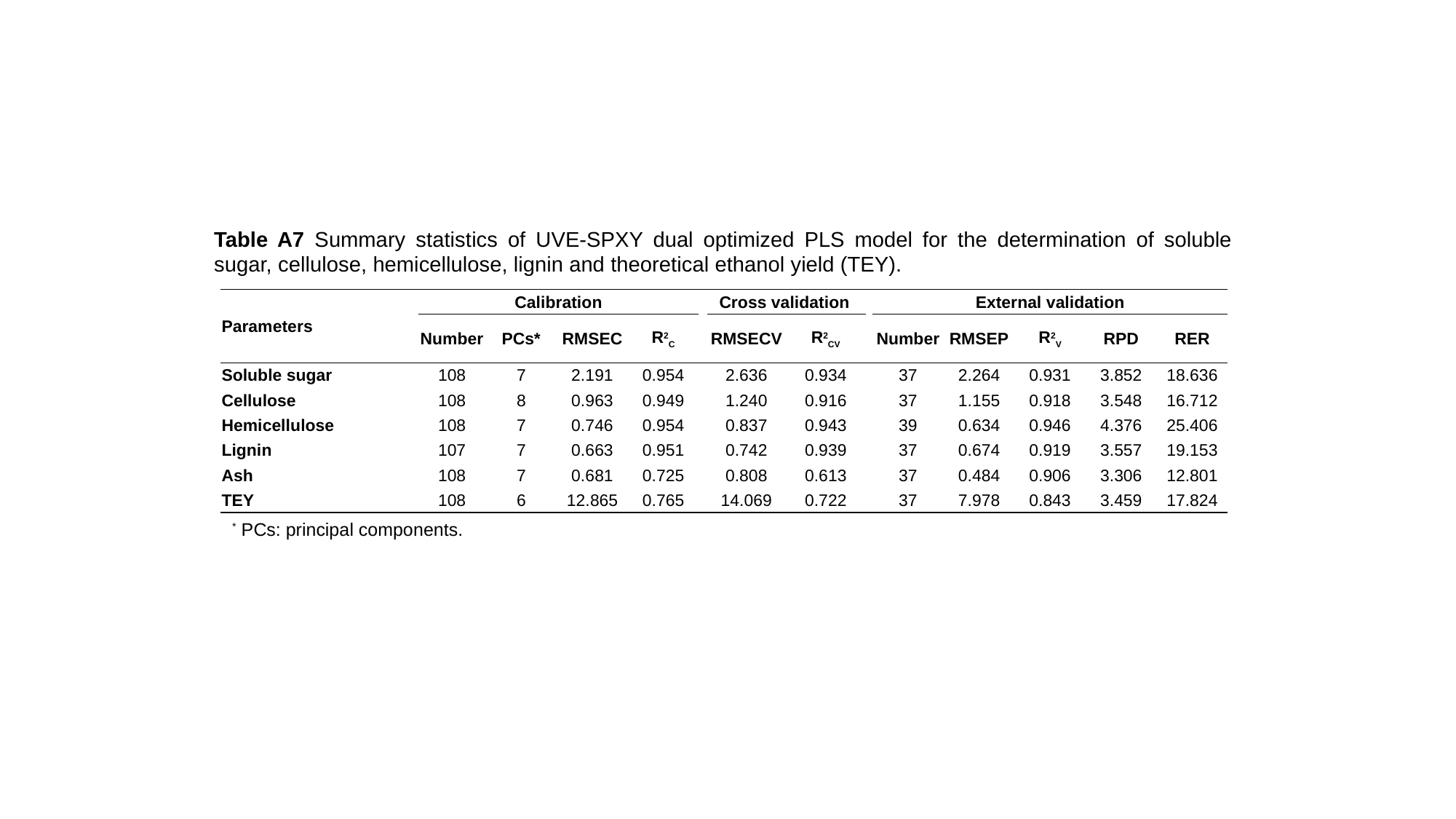

Table A7 Summary statistics of UVE-SPXY dual optimized PLS model for the determination of soluble sugar, cellulose, hemicellulose, lignin and theoretical ethanol yield (TEY).
| Parameters | Calibration | | | | | Cross validation | | | External validation | | | | |
| --- | --- | --- | --- | --- | --- | --- | --- | --- | --- | --- | --- | --- | --- |
| | Number | PCs\* | RMSEC | R2C | | RMSECV | R2CV | | Number | RMSEP | R2V | RPD | RER |
| Soluble sugar | 108 | 7 | 2.191 | 0.954 | | 2.636 | 0.934 | | 37 | 2.264 | 0.931 | 3.852 | 18.636 |
| Cellulose | 108 | 8 | 0.963 | 0.949 | | 1.240 | 0.916 | | 37 | 1.155 | 0.918 | 3.548 | 16.712 |
| Hemicellulose | 108 | 7 | 0.746 | 0.954 | | 0.837 | 0.943 | | 39 | 0.634 | 0.946 | 4.376 | 25.406 |
| Lignin | 107 | 7 | 0.663 | 0.951 | | 0.742 | 0.939 | | 37 | 0.674 | 0.919 | 3.557 | 19.153 |
| Ash | 108 | 7 | 0.681 | 0.725 | | 0.808 | 0.613 | | 37 | 0.484 | 0.906 | 3.306 | 12.801 |
| TEY | 108 | 6 | 12.865 | 0.765 | | 14.069 | 0.722 | | 37 | 7.978 | 0.843 | 3.459 | 17.824 |
* PCs: principal components.
