# Supplementary material for: High-throughput analysis of chemical components and theoretical ethanol yield of dedicated bioenergy sorghum using dual-optimized partial least squares calibration models
Source: Biotechnol Biofuels. 2017 Sep 4;10:206. doi: 10.1186/s13068-017-0892-z (PMC5584014; doi:10.1186/s13068-017-0892-z)
Supplement: Supplementary file 2 — Additional file 2: Figure A1. Plots of predicted versus measured values of parameters. Soluble sugar (a), cellulose (b), hemicellulose (c), lignin (d), ash (e), and theoretical ethanol yield (f) for the external validation subsets based on CARS-SPXY dual-optimized PLS models. The ${\text{R}}_{\text{V}}^{2}$RV2 represents the square of the correlation coefficients of the external validation subsets. Figure A2. Plots of predicted versus measured value of parameters. Soluble sugar (a), cellulose (b), hemicellulose (c), lignin (d), ash (e), and theoretical ethanol yield (f) for the external validation subsets based on SR-SPXY dual-optimized PLS models. The ${\text{R}}_{\text{V}}^{2}$RV2 represents the square of the correlation coefficients of the external validation subsets. Figure A3. Plots of predicted versus measured value of parameters. Soluble sugar (a), cellulose (b), hemicellulose (c), lignin (d), ash (e), and theoretical ethanol yield (f) for the external validation subsets based on VIP-SPXY dual-optimized PLS models. The ${\text{R}}_{\text{V}}^{2}$RV2 represents the square of the correlation coefficients of the external validation subsets. Figure A4. Plots of predicted versus measured value of parameters. Soluble sugar (a), cellulose (b), hemi [file 13068_2017_892_MOESM2_ESM.pptx]

## Slide 1
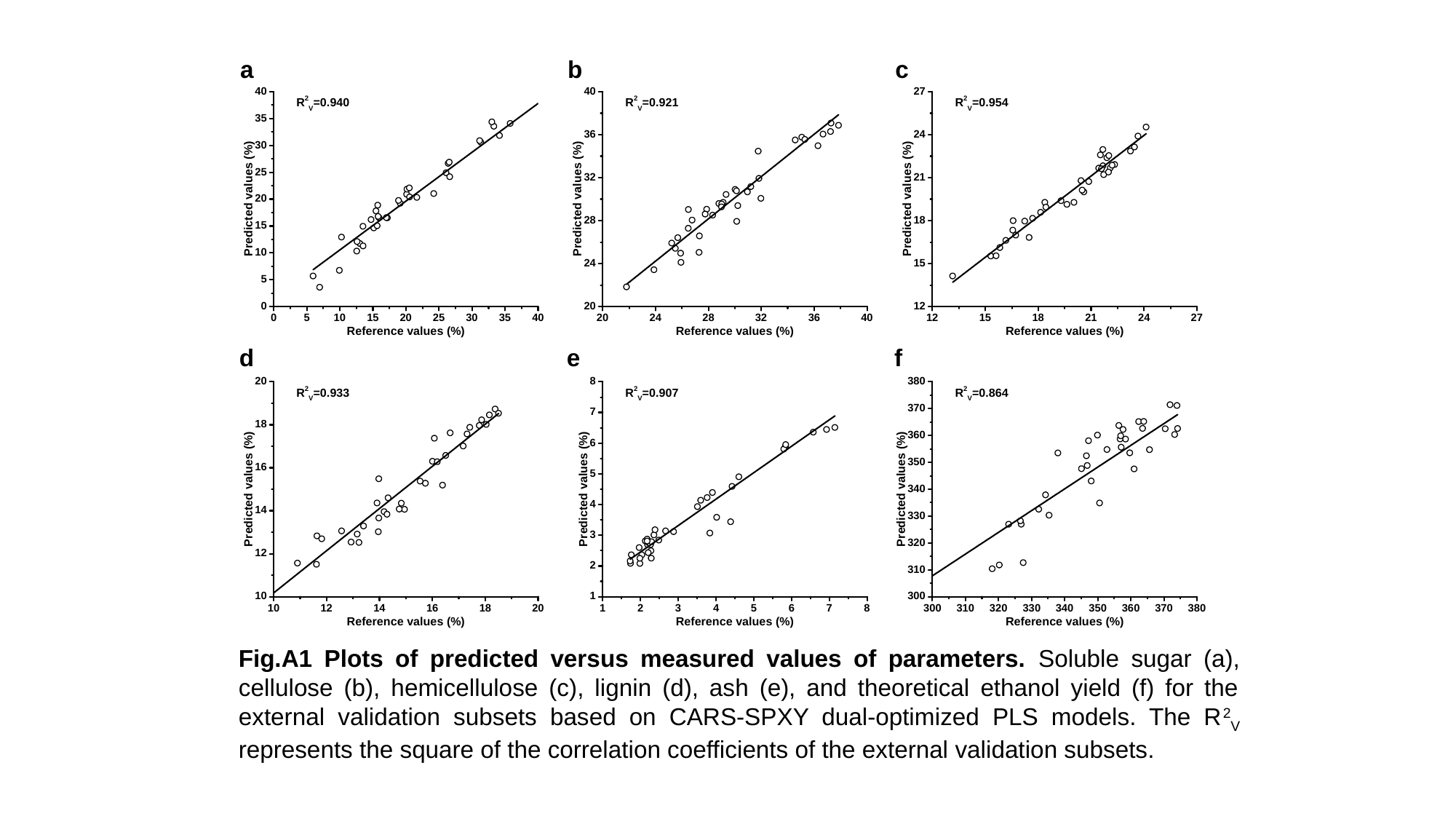

a
b
c
d
e
f
Fig.A1 Plots of predicted versus measured values of parameters. Soluble sugar (a), cellulose (b), hemicellulose (c), lignin (d), ash (e), and theoretical ethanol yield (f) for the external validation subsets based on CARS-SPXY dual-optimized PLS models. The R2V represents the square of the correlation coefficients of the external validation subsets.

## Slide 2
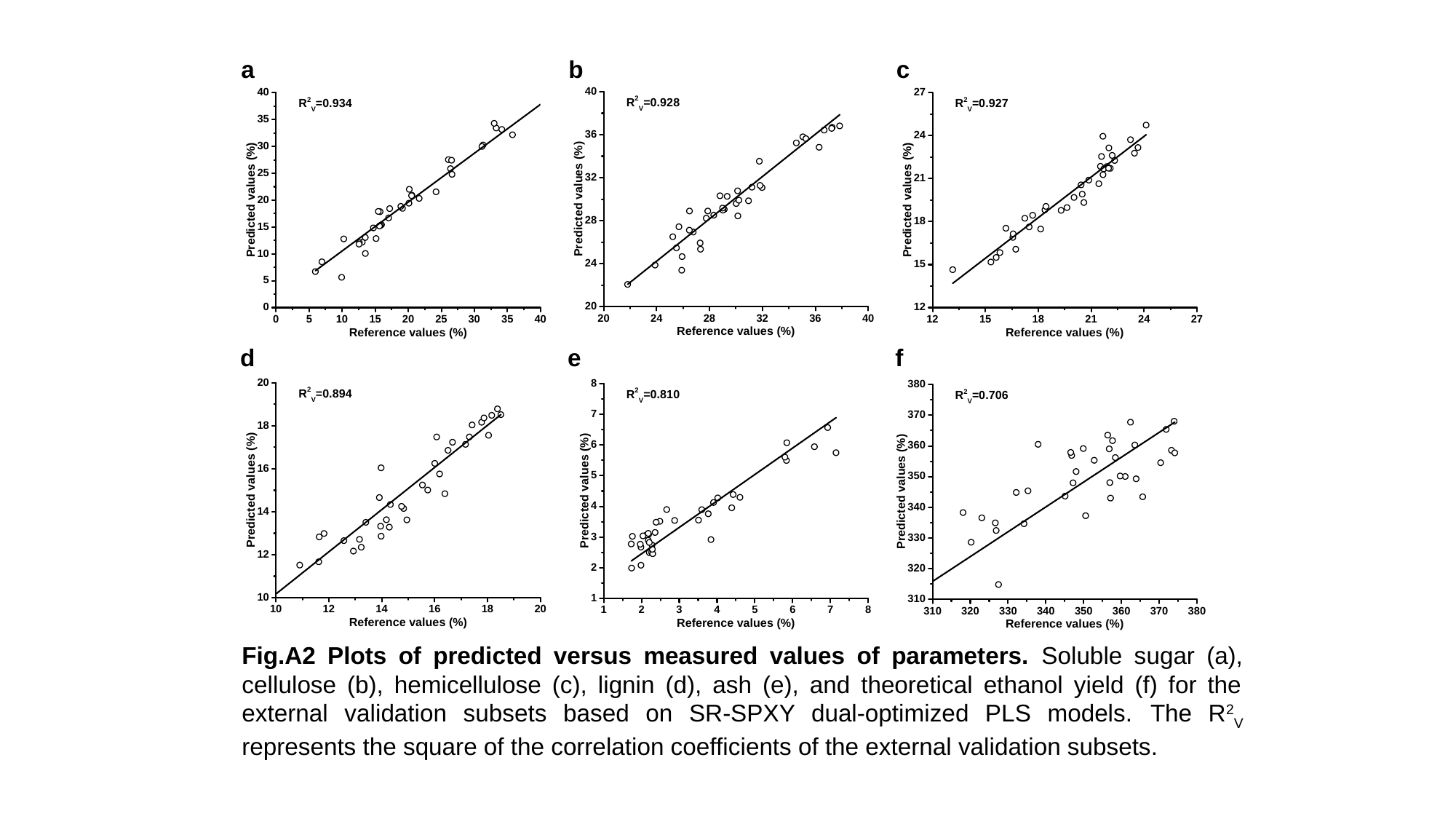

a
b
c
d
e
f
Fig.A2 Plots of predicted versus measured values of parameters. Soluble sugar (a), cellulose (b), hemicellulose (c), lignin (d), ash (e), and theoretical ethanol yield (f) for the external validation subsets based on SR-SPXY dual-optimized PLS models. The R2V represents the square of the correlation coefficients of the external validation subsets.

## Slide 3
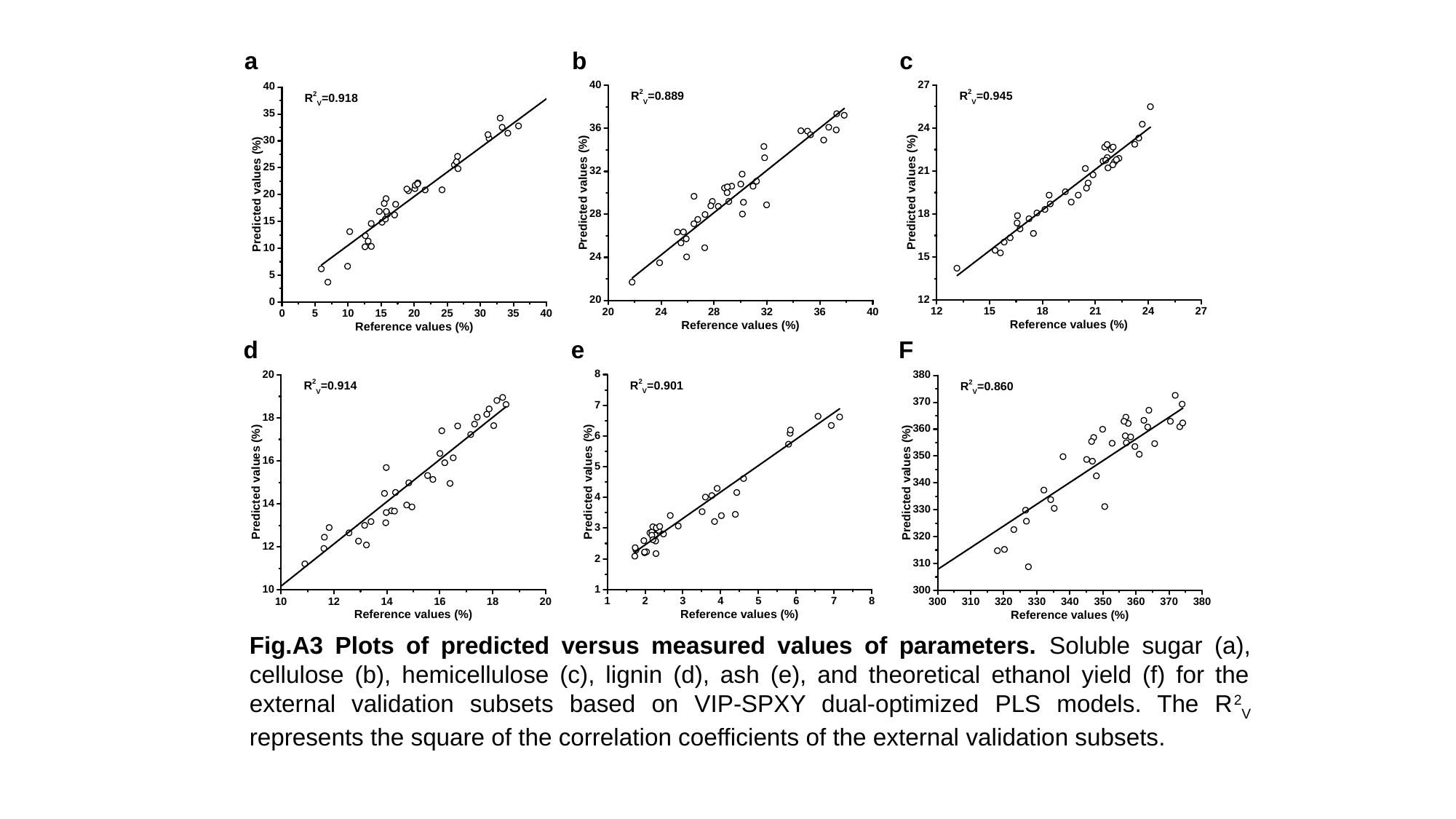

a
b
c
d
e
F
Fig.A3 Plots of predicted versus measured values of parameters. Soluble sugar (a), cellulose (b), hemicellulose (c), lignin (d), ash (e), and theoretical ethanol yield (f) for the external validation subsets based on VIP-SPXY dual-optimized PLS models. The R2V represents the square of the correlation coefficients of the external validation subsets.

## Slide 4
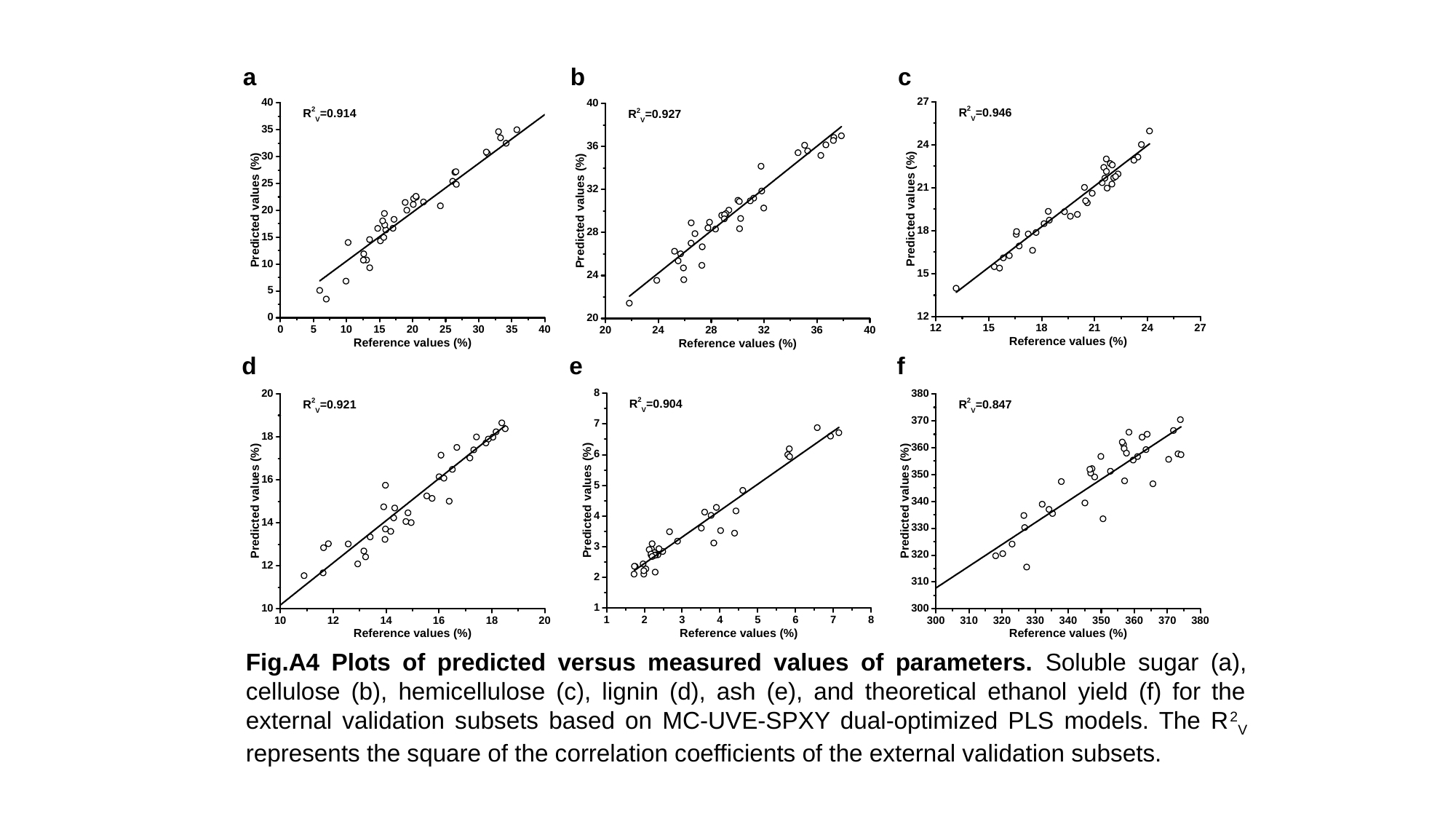

a
b
c
d
e
f
Fig.A4 Plots of predicted versus measured values of parameters. Soluble sugar (a), cellulose (b), hemicellulose (c), lignin (d), ash (e), and theoretical ethanol yield (f) for the external validation subsets based on MC-UVE-SPXY dual-optimized PLS models. The R2V represents the square of the correlation coefficients of the external validation subsets.

## Slide 5
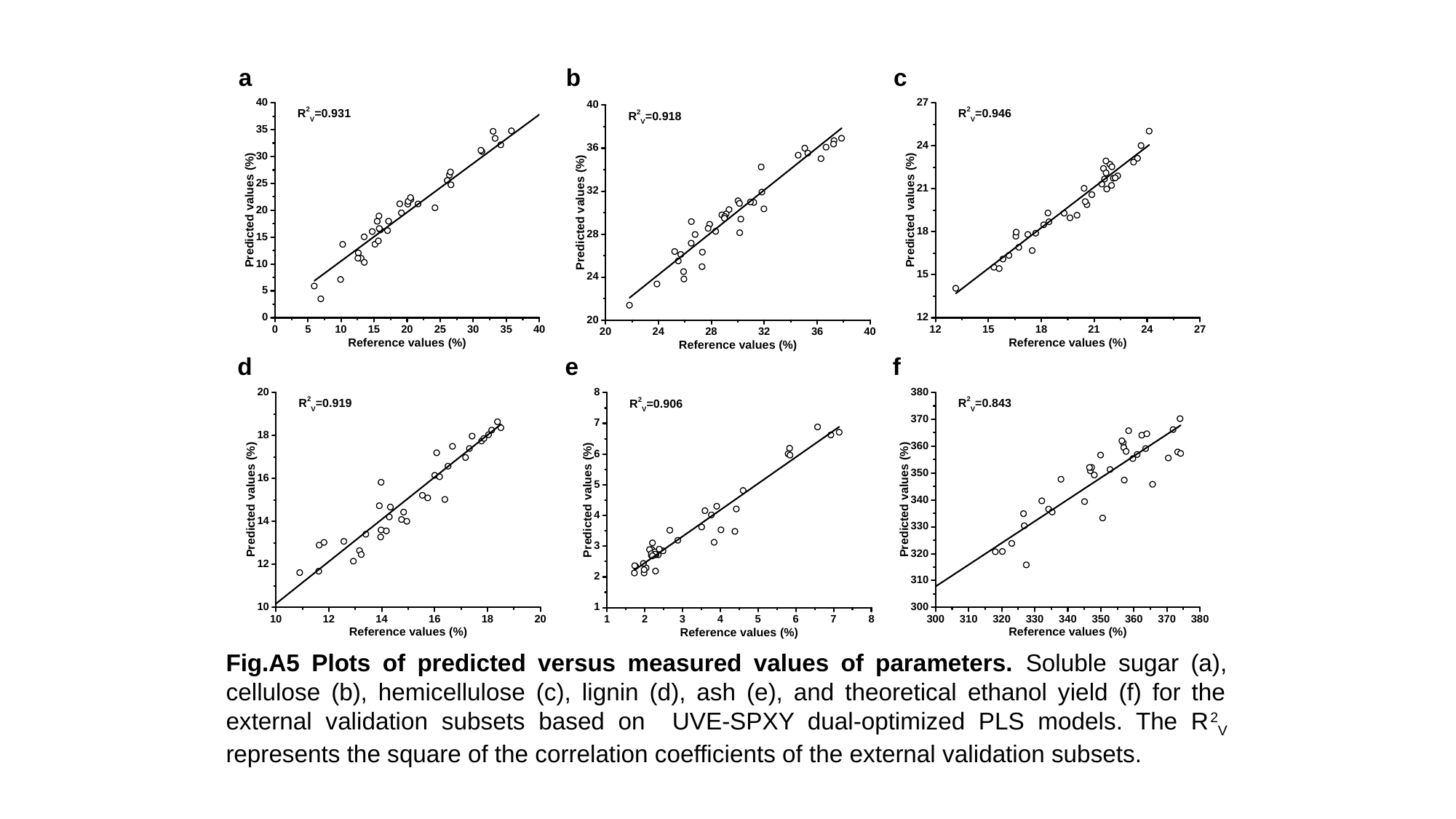

a
b
c
d
e
f
Fig.A5 Plots of predicted versus measured values of parameters. Soluble sugar (a), cellulose (b), hemicellulose (c), lignin (d), ash (e), and theoretical ethanol yield (f) for the external validation subsets based on UVE-SPXY dual-optimized PLS models. The R2V represents the square of the correlation coefficients of the external validation subsets.
